# An application of artificial neural network (ANN) for comparative performance assessment of solar chimney (SC) plant for green energy production

**DOI:** 10.1038/s41598-023-46505-1

**Published:** 2024-01-10

**Authors:** Dipak Kumar Mandal, Nirmalendu Biswas, Nirmal K. Manna, Dilip Kumar Gayen, Ali Cemal Benim

**Affiliations:** 1 Department of Mechanical Engineering, Government Engineering College, Samastipur, Bihar 848127 India; 2 Department of Power Engineering, Jadavpur University, Salt Lake, Kolkata, 700106 India; 3 Department of Mechanical Engineering, Jadavpur University, Kolkata, 700032 India; 4 Department of Computer Science Engineering, College of Engineering and Management, Kolaghat, 721171 India; 5 Department of Mechanical and Process Engineering, Düsseldorf University of Applied Sciences, Düsseldorf, Germany

**Keywords:** Energy science and technology, Engineering

## Abstract

This study aims to optimize the power generation of a conventional Manzanares solar chimney (SC) plant through strategic modifications to the collector inlet height, chimney diameter, and chimney divergence. Employing a finite volume-based solver for numerical analysis, we systematically scrutinize influential geometric parameters, including collector height (*h*_i_ = 1.85 to 0.1 m), chimney inlet diameter (*d*_ch_ = 10.16 to 55.88 m), and chimney outlet diameter (*d*_o_ = 10.16 to 30.48 m). Our findings demonstrate that reducing the collector inlet height consistently leads to increased power output. The optimal collector inlet height of *h*_i_ = 0.2 m results in a significant power increase from 51 to 117.42 kW (~ 2.3 times) without additional installation costs, accompanied by an efficiency of 0.25%. Conversely, enlarging the chimney diameter decreases the chimney base velocity and suction pressure. However, as turbine-driven power generation rises, the flow becomes stagnant beyond a chimney diameter of 45.72 m. At this point, power generation reaches 209 kW, nearly four times greater than the Manzanares plant, with an efficiency of 0.44%. Nevertheless, the cost of expanding the chimney diameter is substantial. Furthermore, the impact of chimney divergence is evident, with power generation, collector efficiency, overall efficiency, and collector inlet velocity all peaking at an outer chimney diameter of 15.24 m (corresponding to an area ratio of 2.25). At this configuration, power generation increases to 75.91 kW, approximately 1.5 times more than the initial design. Remarkably, at a low collector inlet height of 0.2 m, combining it with a chimney diameter of 4.5 times the chimney inlet diameter (4.5*d*_ch_) results in an impressive power output of 635.02 kW, signifying a substantial 12.45-fold increase. To model the performance under these diverse conditions, an artificial neural network (ANN) is effectively utilized.

## Introduction

A solar chimney (SC) power plant is a device designed for harnessing solar energy to generate power. It consists of three primary components: the collector absorber plate, a transparent cover, and a chimney. Energy conversion takes place from thermal to kinetic energy and finally into electricity^1–4^. Despite the simplicity in construction and principle, several issues exist, mostly the low efficiency of the plant. Apart from the material of the transparent cover and absorber plate, geometric parameters like collector diameter, collector inlet, chimney height, chimney diameter, and divergence influence the performance^5^. So far, the first plant is reported in Spain (Manazaranes) by Haaf et al.^6,7^ in 1982 for generating ~ 50 kW power. The study on the variation of collector inlet (Table 1), variation of chimney diameter (Table 2), and divergence (Table 3) are listed in detail.

**Table 1:** Impact of collector inlet heights (*h*_*i*_) on the performance of SC plant.

Author	Studied parameters	Observations	Remarks
Kasaeian et al.^8^	Numerical and analytical study on the effect of collector inlets of 6, 8, and 12 cm on velocity and temperature	6 cm collector through showed maximum velocity and temperature	No optimum entrance value is studied
Patel et al.^9^	Collector inlet height varies from 0.05 and 0.2 m in the numerical study	The opening of 0.05 m is the best choice	No optimum value is studied
Vieira et al.^10^	Numerical work to investigate the effect of collector inlet	No significant effect has been noted	No question of optimum value
Ghalamchi et al.^11^	Collector entrance length varied for 0.04, 0.06, 0.1 and 0.14 m, experimentally	0.06 m shows the maximum velocity	The optimum condition is not studied
Toghraie et al.^12^	Numerical study varying collector inlet from 1 to 10 m on efficiency and power	Power and efficiency are high at low inlet height	No optimum height is investigated
Yapici et al.^13^	Inlet height is varied from 4 to 10 cm, numerical work	The optimal dimension for peak performance is 6 cm	–
Golzardi et al.^14^	Experimental assessment by varying collector entrances	In one geometry, decrease in collector entrance gains exit velocity (17%) and energy transfer (62%). Other geometry by dropping 16 to 8 cm results 13% and 37% improvement	No optimum value is investigated
Mandal et al.^2^	Numerical as well as experimental investigation for performance	Effective at some value of inlet height	Optimum value is noted

**Table 2:** Impact of chimney diameter (*d*_*ch*_) on the performance of SC plant.

Author	Studied parameters	Observations	Remarks
Ming et al.^15^	Numerical study to find best chimney shape	Cylindrical chimney is the best choice. Optimum slenderness ratio ranges from 6 to 8	–
Kasaeian et al.^8^	Numerical and analytical study, velocity measurement for chimney diameters of 10, 20 and 30 cm	The chimney diameter is having more influence than the chimney height. 30 cm diameter chimney is better performer	No optimum value of diameter is studied
Ghalamchi et al.^11^	Studied chimney diameters are 0.1, 0.2 and 0.3 m	Maximum velocity is at 0.1 m	Optimum diameter is not studied
Toghraie et al.^12^	Numerical study by varying chimney diameter from 1 to 12 m on mass flow, pressure, and power	Maximum mass flow, power and minimum pressure is noted at optimum chimney diameter of 5 m	–
Cottam et al.^16^	Effect on power generation for chimney radius from 50 to 200 m	Optimum chimney radius is 165 m, where the power is maximum	–
Yapici et al.^13^	Numerical investigation by varying chimney diameters (15 to 35 cm)	Optimum chimney diameter is 30 cm to obtain maximum impact on the performance. Further rise in diameter impacts negatively	–

**Table 3:** Impact of chimney divergence (*d*_*o*_) on the performance of SC plant.

Author	Studied parameters	Observations	Remarks
Koonsrisuk and Chitsomboon^17^	Numerical study, studied effect of chimney outlet flow area	Chimney divergence improves the mass flow as well as power	–
Patel et al.^9^	Studied numerically chimney divergence 0-3º	2° is the best configuration	–
Okada et al.^18^	Improvement of power by diffuser tower for 4º divergence angle	Improve power is about 3 times	No optimum value was studied
Vieira et al.^10^	Numerical work to examine the effect of chimney outlet diameter	Showed the improvement of power	No optimum value was studied
Ohya et al.^19^	The use of diffuser-type chimney over cylindrical one is studied numerically and experimentally both	4° shows the optimum value, and the maximum updraft	–
Hu et al.^20^	Effect of Chimney divergence by area ratio is studied upto 32	Area ratio ~ 10 showed the optimum choice of power generation	–
Hassan et al.^21^	Numerical study by Manzaranes plant, effect of divergent chimney, angle 1to 3º	Optimum divergence is 1°, enhancement in velocity 27%, estimated power rise 108%	–
Nasraoui et al.^22^	Conical chimney angle for 0, 3, 6 and 9° is studied numerically	Performance drops after 3°	–
Yapici et al.^13^	Numerical study for different shaped chimney	Divergent chimney is the best for performance	No optimum divergence is studied
Das and Chandramohan^23^	Numerical study to estimate the flow and performance parameters for divergent chimney for divergent of 1 to 5º	Optimum divergence is 2°, enhancement in velocity 59%, power 280% and efficiency by 242%	–
Das and Chandramohan^24^	Performance characteristics for different divergent chimneys numerically, divergent angle varies 1 to 5º	Optimum divergence is 2°, enhancement in velocity 59%, pressure 158%, power 311% and efficiency 287%	–
Cuce et al.^25^	Simulation on Manzaranes plant to find the effect of convergent and divergent chimney, area ratio varies from 0 to 10	Optimum area ratio is 4, efficiency rises from 0.29% to 0.83%, and power rises 54.3 to 168.5 kW	–

The above study of collector inlet variation envisages that the flow velocity rises but the flow volume drops for the reduction of inlet height. Several studies have mentioned the rise in power for a small inlet. However, a very small height weakens the flow and, consequently power generation. Very few of the studies have addressed optimum inlet height; this height is different for different sizes of plants. From the effect of chimney diameter, it can be claimed by a few studies that velocity rises for small chimney diameters and power rises, as power is proportional to velocity. Most of the researchers have addressed the rise in power with a rise in chimney diameter. This augments suction pressure and flow volume. However, a very high chimney diameter chokes the flow, as mentioned by a few studies. The optimum diameter depends on chimney height, measured by the slenderness ratio. Its value is 5–6^26^, 6–8^27^. Balijepalli et al.^28^ have mentioned the significance of the chimney height to collector diameter (*d*_*g*_) ratio since the performance of the plant depends on this ratio. Many of the studies have been carried out without the optimum value of diameter. The divergence study claims that suction rises but there is also an optimum divergence angle or area ratio. This is different for different models, any new models require this study. Azad et al.^29^ have worked numerically towards the optimization of design parameters (collector height and diameter, chimney height and diameter) of solar chimneys with desalination plants by using neural networks for power generation and freshwater production. The numerical work of Xu et al.^30^ shows an improvement in power generation up to 120kW by incorporating an energy storage layer for the Spanish prototype. Zhou^31^ has studied the thermal performance of curved sloped collectors on two segmental slopes and compared it with two linearized rising slopes.

The construction of the Manzaranes plant was an inspiration to start innovative research. Considering this plant, the chimney height and how it influences the plant performance for increasing chimney height is investigated by many researchers^32–35^. The optimal chimney height lengthens as the collector diameter rises^36^. The impact of collector slant and diameter is also investigated^32,37–42^. A sloped collector enhances the performance of the plant apart from the collector diameter. The collector roughness is also scrutinized to obtain better effectiveness^43,44^. Few studies, considering heat storage^45^, using radiation and real model^46–48^, using chimney entry fillets^49^, and 2D study^50–52^ have also been noted for the same plant.

Numerous researchers have dedicated their efforts to advancing cleaner energy production, with a particular focus on enhancing the performance of solar chimney (SC) plants. While extensive work has been conducted within the field, the review of existing literature reveals a noticeable gap in the optimization of collector inlet heights. Few researchers have explored this avenue, despite its potential impact on SC plant performance. On the other hand, investigations regarding the optimization of chimney diameter and divergence have received greater attention, primarily to maximize power generation^4,14,29–31^. However, when it comes to the classical Manzanares SC plant, the examination of optimal collector inlet height and chimney diameter remains notably limited. This uncharted territory within the realm of thermo-fluid flow phenomena within the SCPP, including collector inlet height and chimney diameter, has provided the impetus for our novel approach, taking on the challenge of improving SC plant performance. Our study is aimed at optimizing the standard Manzanares SC plant by altering the collector inlet (*h*_i_), chimney diameter (*d*_ch_), and chimney divergence based on the exit diameter (*d*_o_). Furthermore, it involves a comparative thermal assessment of the Manzanares SC plant under various geometric constraints, spanning key parameters such as collector inlet (*h*_i_ = 1.85 to 0.1 m), chimney inlet diameter (10.16 to 55.88 m or 1.0*d*_ch_ to 5.5*d*_ch_), and chimney outlet diameter (10.16 to 30.48 m or 1.0*d*_ch_ to 3*d*_ch_). The outcomes of our analysis are presented in the form of fluid flow and temperature distributions, encompassing pressure, velocity, temperature, and mass flow, as well as performance parameters including power and efficiencies. Additionally, we have endeavored to construct a performance model using an artificial neural network (ANN), a valuable tool that can greatly assist designers in the creation of prototypes.

## Methodology and analysis

### Formation of physical domain

**Figure 1:**
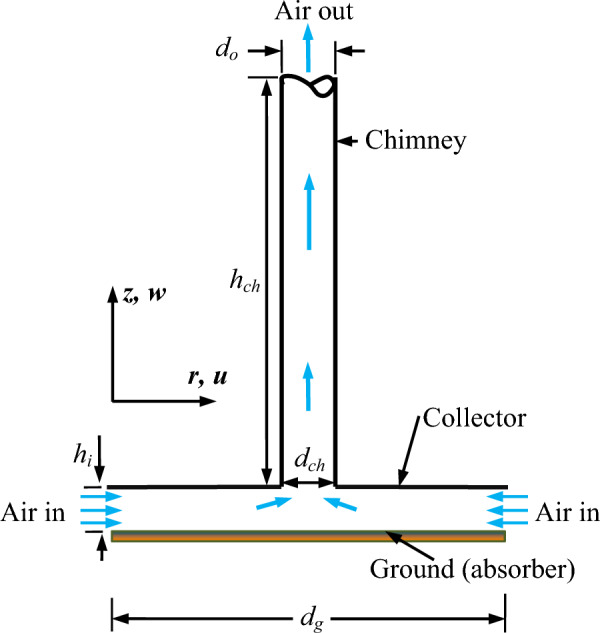
Schematic of the SC model for cylindrical chimney.

In the present analysis, the well-known Manzanares unit^6^ is selected as the basic model as depicted in Fig. 1. For the investigation of the SC model, it is supposed that the ambient air is incompressible and surrounding ambient conditions have no change with time. Here, the density variation at low temperatures is taken care of by following the Boussinesq method. Furthermore, the solar radiation of 10^3^ W/m^2^ is supposed to be constant during each process^53^.

### Governing equations and modeling

The transport equations for the flow model of the SC plant consist of mass, momentum, and energy conservation equations, which are solved numerically by applying the proper boundary conditions. These equations^54, 55^ are presented (in the tensor form) as:

1 $$\frac{\partial }{{\partial x_{i} }}\left( {\rho u_{i} } \right) = 0,$$

2 $$\frac{\partial }{{\partial x_{j} }}\left( {\rho u_{i} u_{j} } \right) = - \frac{\partial p}{{\partial x_{i} }} + \frac{\partial }{{\partial x_{j} }}\left[ {(\mu_{t} + \mu )\left( {\frac{{\partial u_{i} }}{{\partial x_{j} }} + \frac{{\partial u_{j} }}{{\partial x_{i} }}} \right) - \frac{2}{3}(\mu_{t} + \mu )\frac{{\partial u_{i} }}{{\partial x_{i} }}\delta_{ij} } \right] + \rho g_{i} \beta \Delta T,$$

3 $$\frac{\partial }{{\partial x_{j} }}\left( {\rho u_{i} T} \right) = \frac{\partial }{{\partial x_{j} }}\left[ {\left( {\frac{\mu }{\Pr } + \frac{{\mu_{t} }}{{\sigma_{t} }}} \right)\frac{\partial T}{{\partial x_{j} }}} \right] + q_{rad} ,$$

where the indices i and j indicate 1, 2, and 3, respectively), Pr and σ_t_ are the Prandtl number and turbulent Prandtl number. The buoyant force is expressed as *ρβg*_*i*_*ΔT* in Eq. (2). The symbols *μ* and *μ*_*t*_ in Eqs. (2) and (3) correspond to the laminar and turbulent viscosity, respectively. The *q*_rad_ stands for radiative heat flux obtained by solving the radiative transfer equation (RTE) given later in Eq. (6). The Kronecker delta is expressed by the symbol *δ* (wherever *δ* = 1, while *i* = *j* and else *δ* = 0).

In the present study, the fluid flow remains in a turbulent flow regime. The RNG $k - \varepsilon$ turbulence model equations^33,56–59^ are given by

4 $$\frac{\partial }{{\partial x_{i} }}\left( {\rho ku_{i} } \right) = \frac{\partial }{{\partial x_{j} }}\left[ {\alpha_{k} \mu_{eff} \frac{\partial k}{{\partial x_{j} }}} \right] + G_{k} + G_{b} + \rho \varepsilon - Y_{M} + S_{k} ,$$

5 $$\frac{\partial }{{\partial x_{i} }}\left( {\rho \varepsilon u_{i} } \right) = \frac{\partial }{{\partial x_{j} }}\left[ {\alpha_{\varepsilon } \mu_{eff} \frac{\partial \varepsilon }{{\partial x_{j} }}} \right] + C_{1\varepsilon } \frac{\varepsilon }{k}\left( {G_{k} + C_{3\varepsilon } G_{b} } \right) - C_{2\varepsilon } \rho \frac{{\varepsilon^{2} }}{k} - R_{\varepsilon } + S_{\varepsilon } .$$

Different terms in RNG $k - \varepsilon$ turbulence model equations are shown in Table 4.

**Table 4:** Different terms in RNG $k - \varepsilon$ turbulence model equations.

Terms	Correlations
Energy production due to mean velocity gradient, ($G_{k}$)	$G_{k} = - \rho \overline{{u^{\prime}_{i} u^{\prime}_{j} }} \frac{{\partial u_{i} }}{{\partial x_{j} }}$
Energy production owing to buoyancy ($G_{b}$)	$G_{b} = \beta g_{i} \frac{{\mu_{t} }}{{\Pr_{t} }}\frac{\partial T}{{\partial x_{i} }}$
The dissipation rate in compressible turbulence, ($Y_{M}$)	$Y_{M} = 2\rho \varepsilon M_{t}^{2}$
Mach number for turbulent flow ($M_{t}$)	$M_{t} = \sqrt {k/\alpha^{2} }$
Additional terms ($R_{\varepsilon }$)	$R_{\varepsilon } = \frac{{C_{\mu } \rho \eta^{3} \left( {1 - \eta /\eta_{0} } \right)}}{{1 + \beta \eta^{3} }}\frac{{\varepsilon^{2} }}{k}$
Thermal diffusivity ($\alpha$)	$\alpha = k/(\rho c_{p} )$

The values of $\eta_{0}$ = 4.38, $\beta$ = 0.012 and $\eta = {{Sk} \mathord{\left/ {\vphantom {{Sk} \varepsilon }} \right. \kern-0pt} \varepsilon }$ are considered^54^.

In the study, the ANSYS-Fluent 18.1 solver, in combination with the validated Discrete Ordinates (DO) model for radiation transport^41,43,58^, was employed. The DO model discretizes angular space into discrete angles, facilitating directional radiation analysis and numerical determination of radiation intensity (*I*). The radiative transfer equation (RTE), as presented in Eq. (6), mathematically describes solar radiation transport within the medium, taking into account phenomena such as absorption, emission, scattering, and phase functions. The RTE predicts the distribution of radiation intensity and the resulting radiative heat flux within the computational domain. This flux serves as a critical source term (q_rad_) in the energy equation, described in Eq. (3), which considers energy transport mechanisms including convection, conduction, and radiation. ANSYS-Fluent seamlessly integrates the RTE, DO model, and their coupling with the energy equation, ensuring accurate radiation simulations within the Solar Chimney Power Plant (SCPP).

The radiative transfer equation is expressed as

6 $$\nabla .\left( {I\left( {\vec{r},\vec{s}} \right)\vec{s}} \right) + \left( {a + \sigma_{s} } \right)I\left( {\vec{r},\vec{s}} \right) = am^{2} \frac{{\sigma T^{4} }}{\pi } + \frac{{\sigma_{s} }}{4\pi }\int\limits_{0}^{4\pi } {I\left( {\vec{r},\vec{s}^{\prime}} \right)} \varphi \left( {\vec{s}.\vec{s}^{\prime}} \right)d\Omega^{\prime},$$

where $\vec{s}^{\prime}$ and $\sigma_{s}$ denote scattering vector and coefficient, $a$ absorption coefficient, *m* refractive index, $\varphi$ phase function, $\Omega^{\prime}$ solid angle, and *I* radiation intensity (in *W/m*^2^).

The impact of different geometric parameters on the effectiveness of the SC plant is examined through power production, collector efficiency, and overall efficiency^4^. Here, the actual power generation is estimated as

7 $$P_{act} = \eta_{t} \times \Delta p \times Q.$$

In the above equation, the symbols $\eta_{t}$ correspond to the turbine efficiency, which is taken as 0.8^41^, $\Delta p$ is the pressure drop in the turbine, which is calculated as the average pressure at the chimney base (CB) $\times$ the pressure drop ratio (the general value is 2/3)^60^.

Airflow at CB, *Q* = chimney area $\times$ air velocity at CB.

8 $${\text{Collector Efficincy (}}\eta_{{\text{c}}} ) = \frac{{\text{Heat utilized}}}{{\text{Energy available by radiation}}} = \frac{{m_{a} c_{p} \left( {T_{CB} - T_{a} } \right)}}{{A_{coll} \times I}},$$

where $m_{a}$ = air flow at CB, kg/s, *C*_*p*_ = specific heat of air, kJ/kgK, $T_{CB}$ = air temperature at CB, K, $A_{coll}$ = collector area, m^2^, $I$ = irradiation, W/m^2^.

9 $${\text{Overall efficiency (}}\eta_{o} {)} = \frac{{\text{Power produced}}}{{\text{Energy available by radiation}}} = \frac{{P_{act} }}{{IA_{coll} }}.$$

## Numerical technique

### Solution methodology

**Figure 2:**
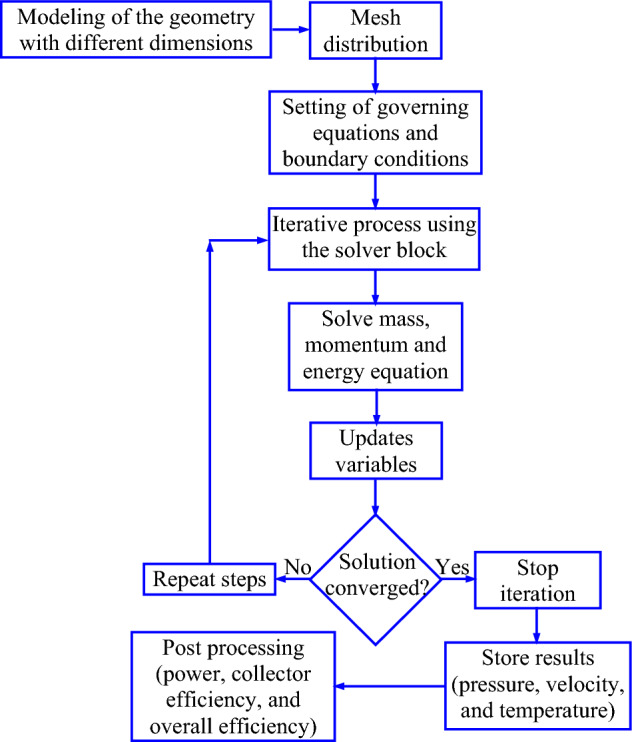
Solution algorithm of the solver in ANSYS fluent.

In this study, the involved transport equations are solved numerically using the finite volume-based ANSYS-Fluent 19.2 solver^59^. The model was generated following the axisymmetric CFD model. Finally, part of the complete model (vertical slice of 15° model) is taken for the computations to reduce the total cost of computations. A similar approach has also been followed by many researchers. For instance, Hassan et al*.*^21^ have considered the 180° model, whereas the 90° and 5° model has been considered by Cuce et al.^25^, and Koonsrisuk and Chitsomboon^17^ respectively. The chosen computational model along with the appropriate boundary conditions are solved in an iterative process applying the SIMPLE algorithm, which couples the pressure and velocity. Thereafter, the second-order upwind scheme is applied to discretize the pressure, momentum, and energy equations. The computational domain is divided into smaller grids. The grids are distributed nonuniformly to capture the correct boundary layers. The maximum *Y* + value in the first cell is taken as 30^38^ and the standard wall function is used in the mesh generation. In general, the finer meshes are distributed adjacent to the solid walls. In order to obtain the converged solution, an iterative process is continued till the reduction of the residuals until 10^–6^ for all the governing equations^61–63^. The corresponding solution procedure is demonstrated in a flow chart, which is shown in Fig. 2.

The adopted boundary conditions for the present study geometry are listed in Table 5. After the mesh independence study, a mesh of 73,862 for the element size 0.80 is taken into consideration for the present analysis. Mesh study is accomplished through different element sizes by comparing the various local parameters such as maximum velocity, temperature, chimney base velocity and temperature, and mass flow rate considering the Manzaranes plant. The considered element sizes taken as 0.90, 0.85, 0.80, 0.75, and corresponding meshes are 53,883 (M1), 62,021 (M2), 73,862 (M3), and 85,551 (M4), respectively. From the comparison, it is observed that the cumulative error level for the different local variables in the consecutive grids decreases with the decreasing element size. Furthermore, the change between element sizes 0.8 and 0.75 is much less. With this comparison, 0.8 element size (M3) is finalized for the extensive study without further increasing computational time for the study. In fact, for obtaining the converged and stable solution under all the parametric variations, a minimum convergence criterion of 10^–6^ for the maximum residuals and the mass defect is chosen for the computation.

**Table 5:** Adopted boundary conditions for present study geometry.

Boundary	Conditions	Magnitudes
Collector	Semitransparent, glass	*h* = 10 W/m^2^K, *T* = 302K
Absorber	Opaque wall	Heat flux, *q* = 0 W/m^2^
Chimney	Adiabatic wall	*q* = 0 W/m^2^
Inlet	Pressure boundary	Zero gauge pressure, *T* = 302K
Outlet	Pressure boundary	Zero gauge pressure

### Validation of present study

**Figure 3:**
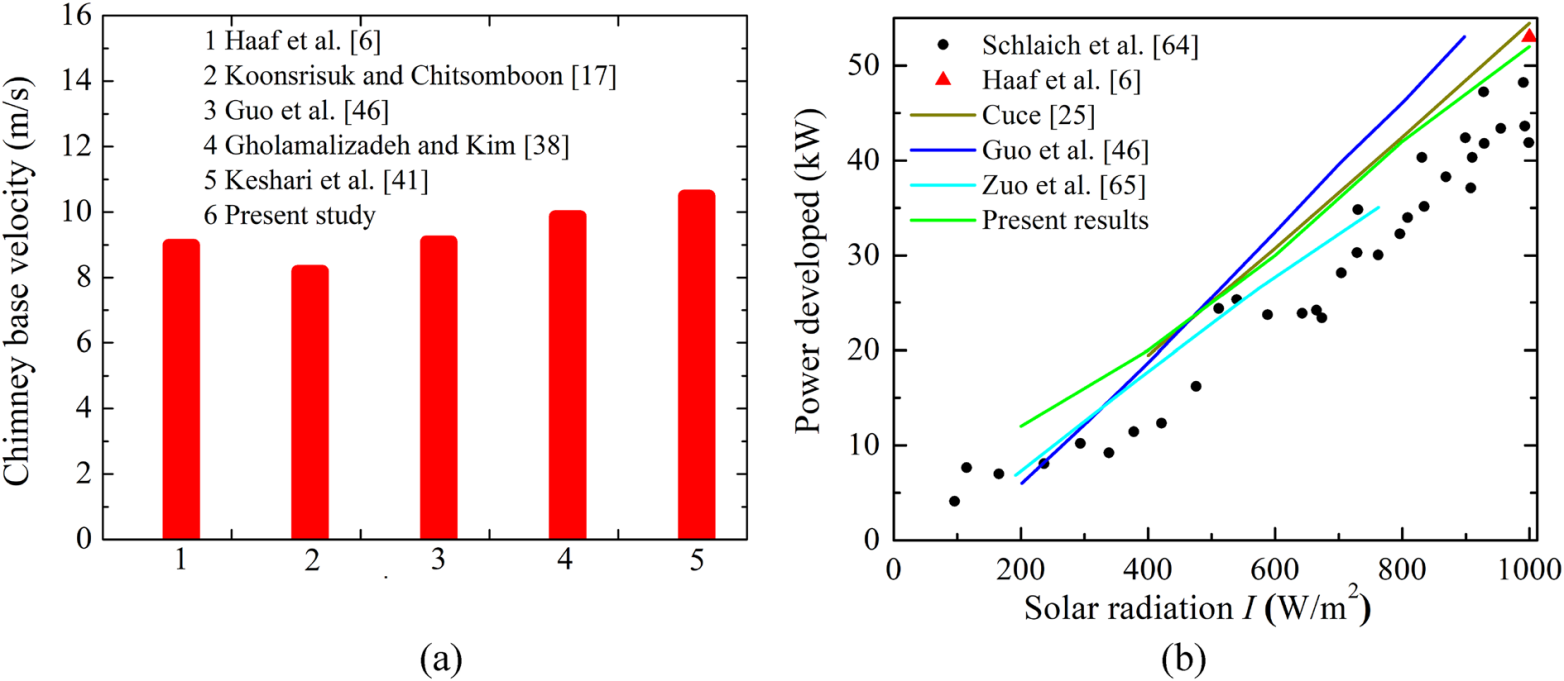
Comparison of (**a**) CB velocities, and (**b**) power produced with published results.

The validation study of the present solver is examined by comparing the CB velocity and power (in kW), as shown in Fig. 3, which confirms the accuracy level of the present solver.

## Discussion of results

This work attempts to enhance the power production of the classical Manzanares SC plant by modifying the collector inlet height, chimney diameter, and its divergence to obtain the best design of the plant. Comparative thermal performance assessment of Manzaranes solar chimney (SC) power plant by different geometries is the main aim of this study. The specified geometries are collector inlet heights (*h*_*i*_), chimney diameter (*d*_*ch*_), and chimney divergence by chimney outlet diameter (*d*_*o*_). The performance assessments are conducted for the range of key controlling geometric parameters like collector height (1.85 to 0.1 m), chimney diameter (10.16 to 55.88 m i.e. 1.0*d*_*ch*_ to 5.5*d*_*ch*_), and chimney outlet diameter (10.16 to 30.48 m i. e., 1.0*d*_*ch*_ to 3.0*d*_*ch*_). The outcomes of the analysis are presented through the fluid flow and temperature distributions (namely pressure, velocity, temperature, and mass flow) and performance parameters (power, collector, and overall efficiency). Furthermore, an attempt has been made to use an artificial neural network (ANN) for developing the generalized performance model, which could be very helpful for the designer for any prototype design.

### Impact of collector inlet heights (h_i_)

The Manzaranes plant uses a collector inlet height of 1.85 m (model, M-1). However, the present study represents the impact of collector inlet height (*h*_*i*_) reduction from 1.85 to 0.1 m systematically, which is defined by the different models, M-1 to M-11 (in Table 6). This analysis computes inlet velocity, chimney base velocity, pressure, temperature, mass flow rate, power produced, collector efficiency, and overall efficiency.

**Table 6:** Models for variations of collector inlet heights (*h*_*i*_).

Model	Collector inlet height (*h*_*i*_)	Chimney diameter (*d*_*ch*_) m	Chimney height (*h*_*ch*_), m	Collector diameter (*d*_*g*_), m	Ratio (*d*_*ch*_/*h*_*i*_))	Ratio *d*_*g*_/*h*_*i*_
M-1	1.85	10.16	194.6	244	5.49	131.9
M-2	1.75	10.16	194.6	244	5.81	139.4
M-3	1.5	10.16	194.6	244	6.77	162.7
M-4	1.25	10.16	194.6	244	8.13	195.2
M-5	1	10.16	194.6	244	10.16	244.0
M-6	0.75	10.16	194.6	244	13.55	325.3
M-7	0.5	10.16	194.6	244	20.32	488.0
M-8	0.25	10.16	194.6	244	40.64	976.0
M-9	0.2	10.16	194.6	244	50.8	1220.0
M-10	0.15	10.16	194.6	244	67.73	1626.7
M-11	0.1	10.16	194.6	244	101.6	2440.0

#### Flow parameters assessment

**Figure 4:**
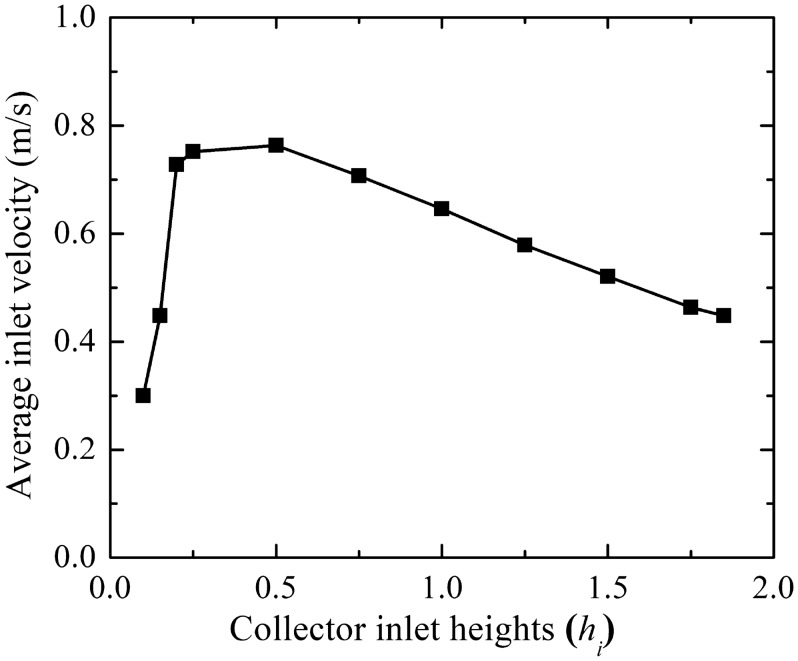
Variation of collector inlet flow velocity at different collector inlet heights (*h*_*i*_).

The induced buoyancy force in-between the collector and absorber plate begins the fluid flow velocity at the collector inlet, the study of the inlet velocity assists the thermo-fluid flow analysis for the plant. The impact of the reduction of collector inlet height (*h*_*i*_) is illustrated in Fig. 4. The reduction of height lessens the effective fluid flow area which in turn reduces the involved fluid flow volume. At lower flow volume, due to higher thermal energy exchange to air, the reduction of collector inlet height grows the inlet fluid velocity. This has been noticed from the collector inlet of 1.85 to 0.2 m (Model, M-1 to M-9). It is mostly owing to the reduction of flow area. Further reduction in the collector inlet height (*h*_*i*_) lowers the magnitude of inlet flow velocity up to model, M-11. The lower flow volume does not extract whole thermal energy and at the same time, the rise in flow velocity raises the viscous force, which is the reason for the dropping of inlet velocity despite decreasing flow area at lower collector inlet height. Therefore, the selection of a collector inlet plays a significant role in improving thermo-fluid flow properties. A reduction of collector inlet height is beneficial but much reduction is not effective. In this Manzaranes plant, the inlet height of 0.2 m (M-9) shows an optimum value for achieving the maximum flow velocity. For the fixed solar irradiation intensity, the absorber area primarily induces the inlet flow velocity at the collector inlet. Therefore, there should be some relationship between collector diameter and collector inlet height, here the value is about 1220 as observed in Table 6.

**Figure 5:**
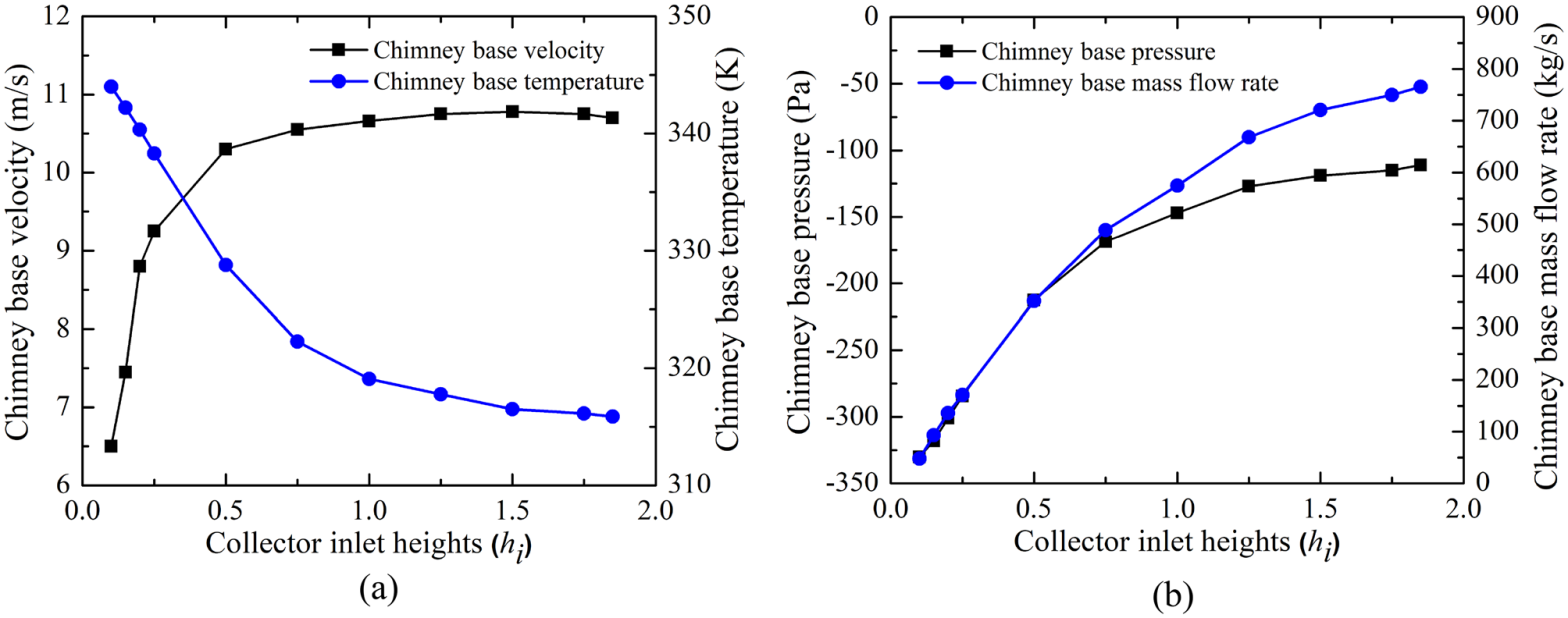
Variations of CB (**a**) velocity and temperature, (**b**) pressure and mass flow at different collector inlet heights (*h*_*i*_).

**Figure 6:**
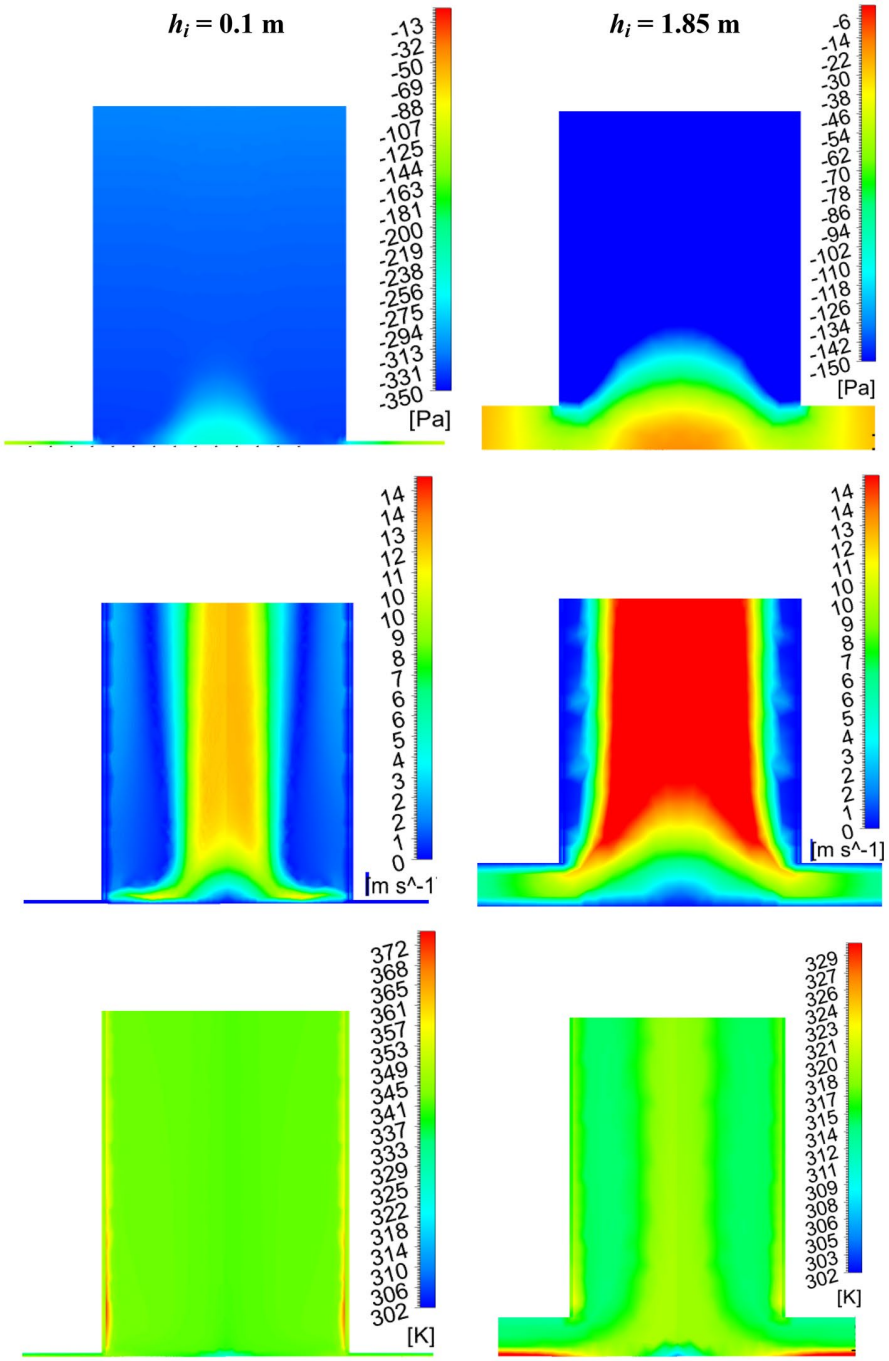
Local pressure, velocity, and temperature contour plots for the variations of collector inlet heights (*h*_*i*_).

The impact of a reduction in collector inlet height (*h*_*i*_) on the CB flow and temperature distribution is illustrated in Fig. 5. The chimney base is taken as a reference to present the flow parameters as the air turbine is installed near the CB. Chimney base velocity drops as the collector inlet height (*h*_*i*_) decreases (in Fig. 5a). The drop in chimney base velocity is sharp after model, M-9. As the chimney area is constant, the volume flow rate through the chimney drops. This drop in the working flow volume in the chimney reduces the collector inlet height (*h*_*i*_). The drop in the flow volume is high after the reduction in the collector inlet for the model, M-9, which is due to the inlet flow velocity drop. The reduction of flow volume gains more thermal energy from the ground plate which results in high temperature as the collector inlet drops. This has been noted in Fig. 5a. With the reduction in flow volume with collector inlet reduction, the suction pressure at the chimney base is noted to rise in Fig. 5b. The temperature of air rises, which in turn reduces the density of air with the reduction of collector inlet. Same time volume flow rate drops, therefore mass flow rate drops as the collector inlet drops (Fig. 5b). The flow contour study by velocity, pressure, and temperature near the CB are illustrated in Fig. 6 for the different models, M-1(*h*_*i*_ = 1.85 m) and M-11 (*h*_*i*_ = 0.1 m). It is evident in the figure, that the chimney base velocity and pressure drop and chimney base temperature rise due to the reduction in the collector inlet (*h*_*i*_).

#### Performance assessment

**Figure 7:**
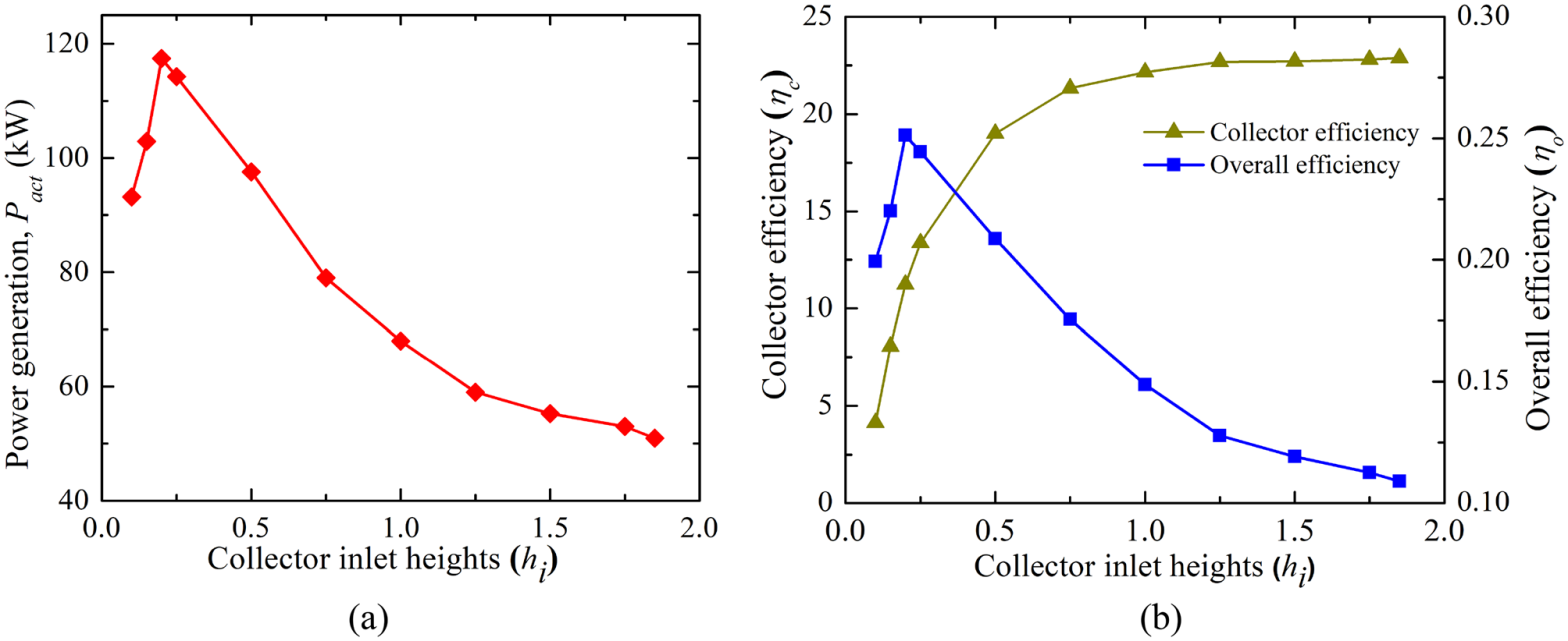
Variation of (**a**) power generation, (**b**) collector efficiency, and overall efficiency at different collector inlet heights (*h*_*i*_).

Under the various geometric parameter variations, the performance study is examined by calculating the power generation and efficiencies (*P*_*act*_, *η*_*c*_, *η*_*o*_), which are shown in Fig. 7. The produced kinetic energy in the air produces turbine power. This turbine work depends on the flow velocity of impinging and pressure drops through the turbine. Previous Sect. “Flow parameters assessment” shows the dropping in the velocity as collector inlet height (*h*_*i*_) drops tends to decrease the power generation by an air turbine. However, the increase in the suction pressure for reduction in the inlet area assists more power generation (*P*_*act*_). The combined effect of velocity and pressure on power generation by the turbine shows the rising tendency of power and the power reaches 117.42 kW at *h*_*i*_ = 0.2 m. This power generation is 2.3 times of the classical Manzaranes plant. Further reduction of *h*_*i*_ lessens the power generated by the turbine (as in Fig. 7a). This can be attributed to that increased power is dominated by the sudden velocity drop in spite of a better pressure drop. Moreover, it can be noted that the power generation by the turbine after *h*_*i*_ = 0.2 m is having greater value than the Manzaranes plant. The collector efficiency (*η*_*c*_) drops with decreasing inlet flow area due to the reduction of mass flow rate despite of increasing temperature of air (as in Fig. 7b). The overall efficiency (*η*_*o*_) shows a similar trend of curve like power (as in Fig. 7b). The efficiency increases to 0.25%. It is also 2.3 times more compared to the Manzaranes plant. Therefore, this exercise clearly shows that collector inlet height (*h*_*i*_) reduction has a positive role in enhanced power generation compared to the classical Manzaranes plant.

The above study summarizes that power developed by the turbine is always higher relative to the Manzaranes plant at less *h*_*i*_. Maximum power lies at *h*_*i*_ = 0.2 m, *P*_*act*_ = 117.42 kW. The maximum efficiency occurs at the same location, which is 0.25%. Therefore, there is no hike in the cost but power rises from 51 to 117.42 kW. As *h*_*i*_ reduces, the chimney base velocity drops, suction pressure increases, the temperature rises, and mass flow drops.

### Impact of chimney diameter (*d*_*ch*_)

The impact of chimney diameter (*d*_*ch*_) is carried out by raising the chimney diameter (*d*_*ch*_) of the Manzaranes plant up to 55.18 m (5.5*d*_*ch*_), details of different models are listed in Table 7 from M-12 to M-20. Like earlier, flow and performance are computed and illustrated subsequently.

**Table 7:** Models in variations of chimney diameters.

Model	Collector inlet (*h*_*i*_)	Chimney diameter (*d*_*ch*_) m	Chimney height (*h*_*ch*_), m	Collector diameter (*d*_*g*_, m	Slenderness ratio (*h*_*ch*_/*d*_*ch*_)	Chimney diametric ratio	*d*_*ch*_/*h*_*i*_
M-1	1.85	10.16	194.6	244	19.15354	1.0	5.49
M-12	1.85	15.24	194.6	244	12.76903	1.5	8.24
M-13	1.85	20.32	194.6	244	9.576772	2.0	10.98
M-14	1.85	25.4	194.6	244	7.661417	2.5	13.73
M-15	1.85	30.48	194.6	244	6.384514	3.0	16.48
M-16	1.85	35.56	194.6	244	5.472441	3.5	19.22
M-17	1.85	40.64	194.6	244	4.788386	4.0	21.97
M-18	1.85	45.72	194.6	244	4.256343	4.5	24.71
M-19	1.85	50.8	194.6	244	3.830709	5.0	27.46
M-20	1.85	55.88	194.6	244	3.482462	5.5	30.21

#### Flow parameters assessment

**Figure 8:**
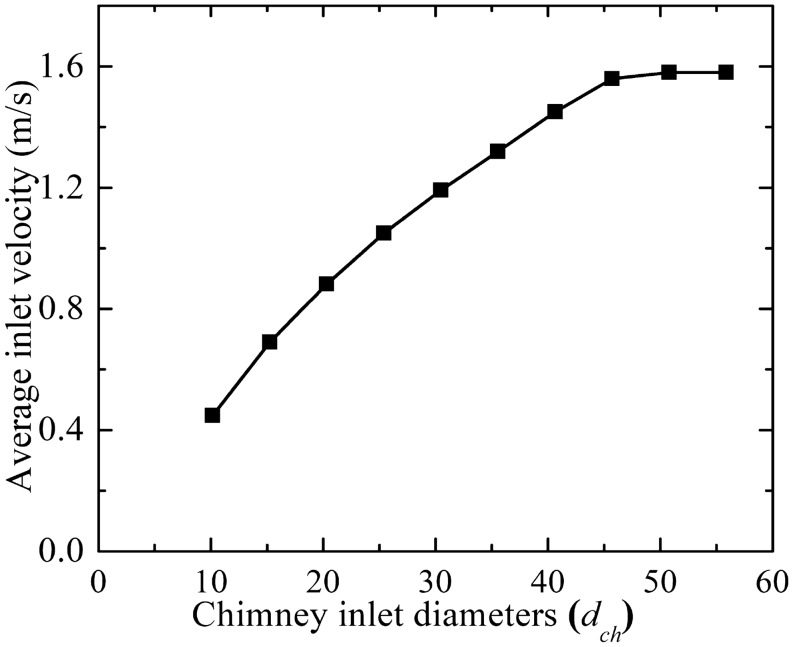
Variation of collector inlet flow velocity for different chimney diameters (*d*_*ch*_).

The increase in the chimney diameter (*d*_*ch*_) shows (as in Fig. 8) the enhancement of inlet collector flow velocity, this means the increase in the flow volume, may be due to the rising in the chimney draft. It is interesting to note that the collector inlet velocity does not rise more after 4.5*d*_*ch*_ (M-18). Further rise in diameter stagnates the flow volume.

**Figure 9:**
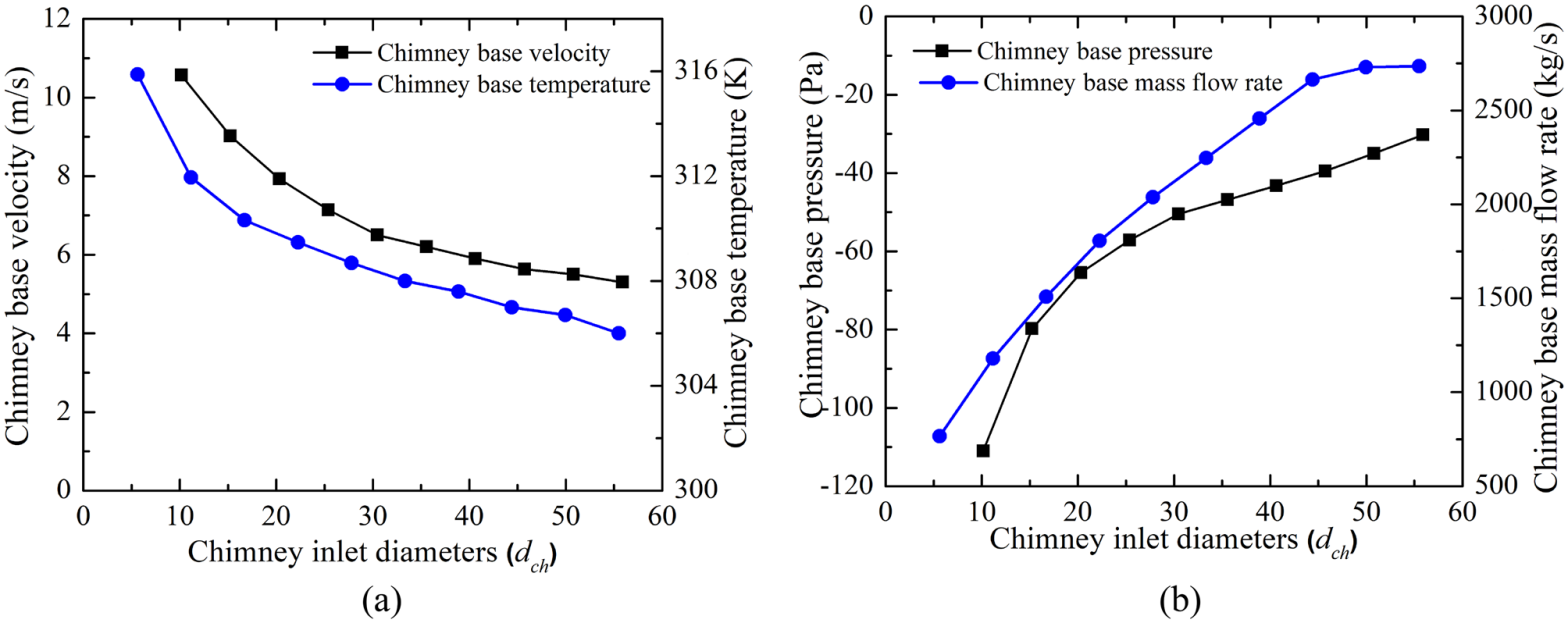
Variations of CB (**a**) velocity and temperature, (**b**) pressure and mass flow at different chimney diameters (*d*_*ch*_).

**Figure 10:**
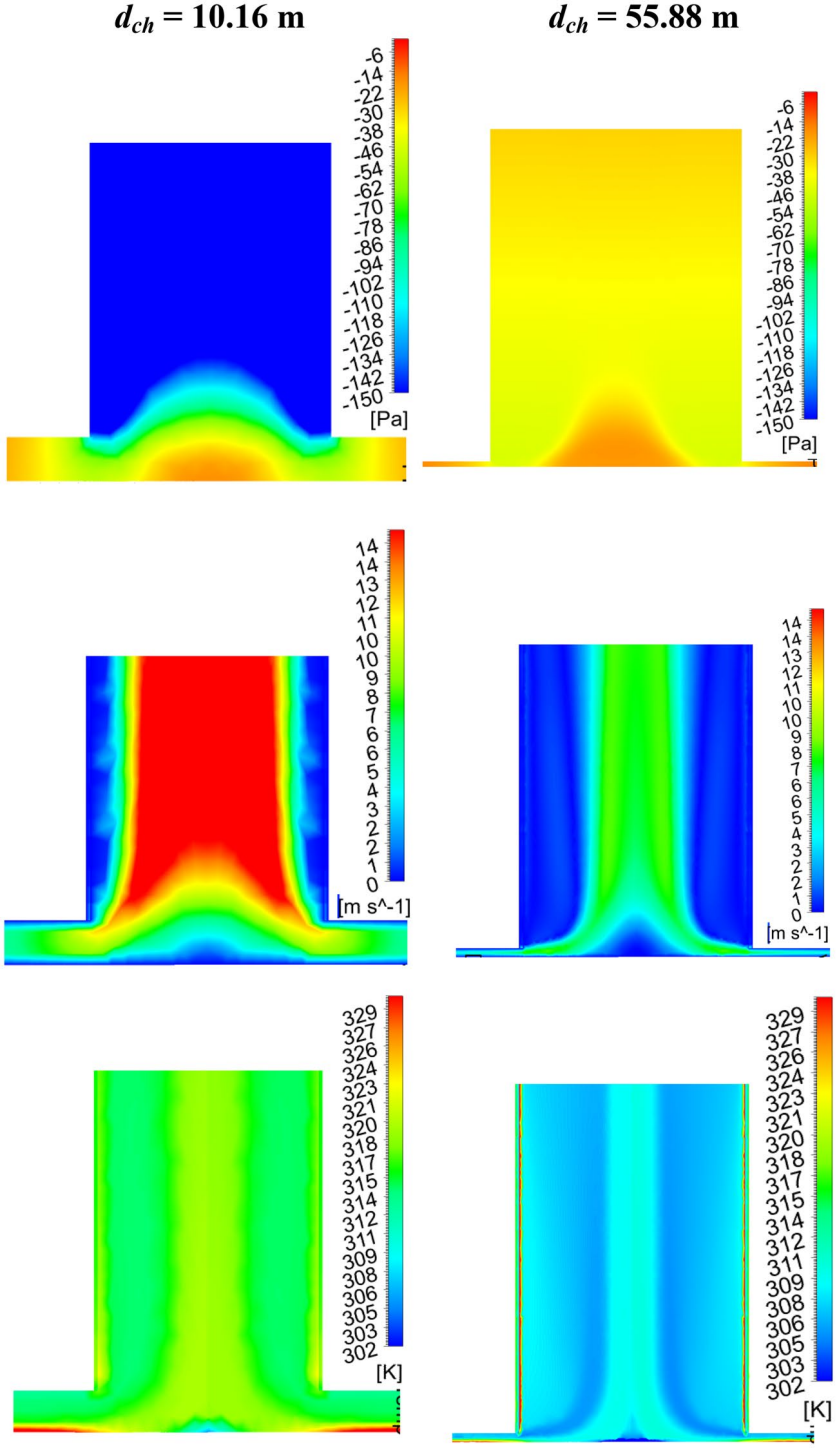
Local pressure, velocity, and temperature contour plots for the variations of chimney diameters.

Figure 9 depicts the drop in the CB velocity (in Fig. 9a) as the chimney diameter enlarges. Here, in spite of the increase in the flow volume of air, velocity lessens, which is due to the rise in the chimney flow area. This high flow volume with rising chimney diameter reduces the temperature of the air. This, in turn, decreases the CB pressure as depicted in Fig. 9b. In spite of a drop in the CB velocity, the mass flow rises because of higher air density and higher flow chimney area. The rise in mass flow rate is not too much after the chimney diameter of 45.72 m (4.5*d*_*ch*_, M-18). The pressure, velocity, and temperature contours at the chimney base (for *d*_*ch*_ of 10.16 m and 55.88 m) in Fig. 10 illustrate a clear understanding of flow features that velocity and temperature drops and suction pressure at the CB decrease for the model, M-20.

#### Performance assessment

**Figure 11:**
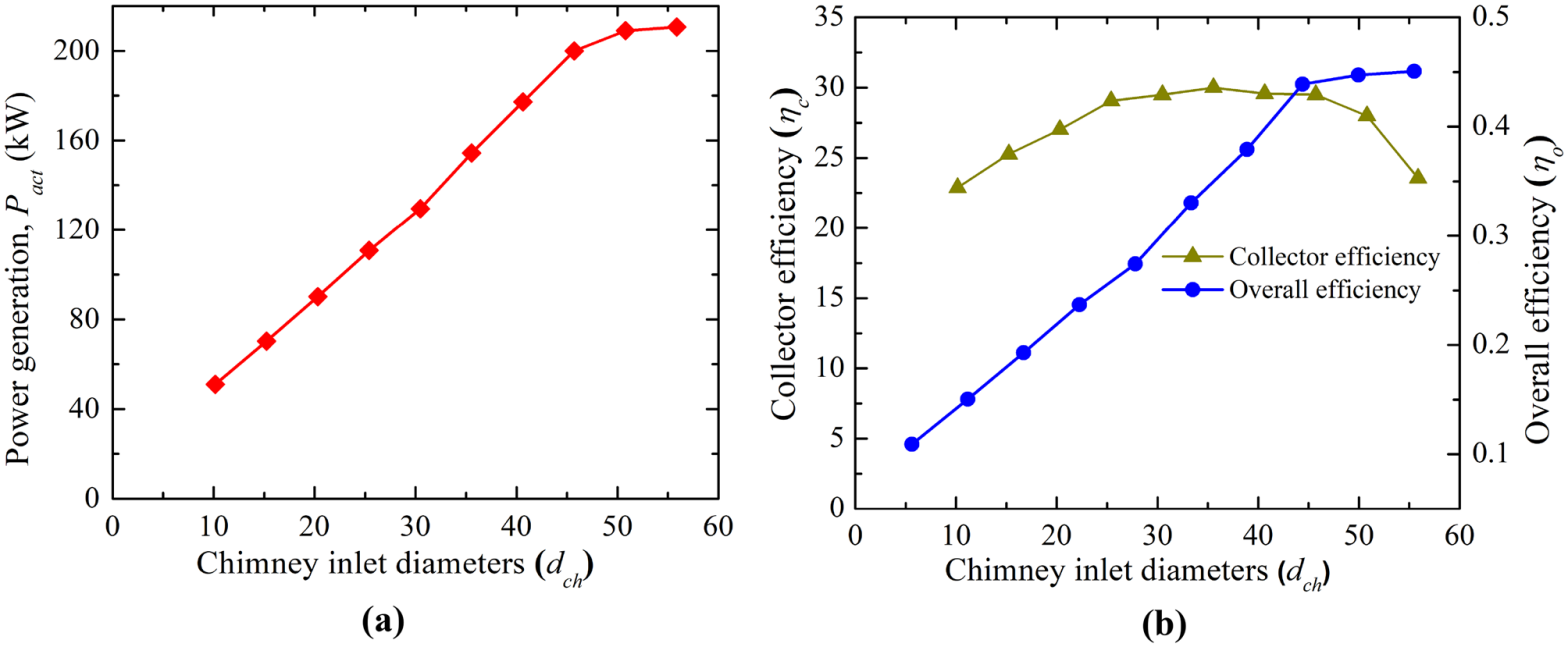
Variation of (**a**) power generation and, (**b**) collector and overall efficiency at different chimney outlet diameters (*d*_*ch*_).

The power generation and efficiencies variation for the different chimney inlet diameter (*d*_*ch*_) is presented in Fig. 11. Earlier, it is depicted that the CB base velocity, and suction pressure both drop as *d*_*ch*_ rises. Both the phenomenon is against the rise in flow energy by the turbine. But the power produced (*P*_*act*_) by the turbine rises as observed from Fig. 11a, which is due to the dominancy of the chimney area, this, in turn, heightens the huge flow volume of air. This enhancement of flow becomes stagnant after the chimney diameter of *d*_*ch*_ = 45.72 m (model, M-18), it results in no significant rise in the power generation further with the chimney diameter. The maximum power generation is* P*_*act*_ = 209 kW which is ~ 4 times than the Manzaranes plant. However, it should be mentioned that some extra cost is required to obtain this increasing diameter of the chimney. The collector efficiency (in Fig. 11b) rises as *d*_*ch*_ rises for the mass flow rate enhancement but this efficiency drops when the mass flow stagnates after some value of chimney diameter. The nature of variation of overall efficiency follows the same trend line as power (in Fig. 11b). The efficiency rises to 0.44% in this case.

From this analysis, it is to be noted that the maximum power available at *d*_*ch*_ = 45.72 m for the slenderness ratio of 4.25. The earlier study depicts the value as 5–6^24^, and 6–8^25^ for the different model plants. The power produced is far more than the power generation at a lower *h*_*i*_ though it involves cost.

### Impact of chimney divergence (*d*_*o*_)

In this section, the effect of chimney divergence (*d*_*o*_) on the performance of the SCPP model is exercised. Here the chimney divergence is made by changing the chimney outlet diameter (*d*_*o*_) as mentioned in Table 8, the chimney inlet diameter (*d*_*ch*_) is made constant of 10.16 m. The variation of models, M-21 to M-24 for the chimney outlet diameter of 30.48 m, which is 3.0*d*_*ch,*_ and the corresponding area ratio of 9.0. A similar flow and performance analysis is carried out to present the comparative results with the reduction of inlet height (*h*_*i*_) and rise in the chimney diameter (*d*_*ch*_).

**Table 8:** Models for the variations of chimney outlet diameters (*d*_*o*_).

Model	Chimney outlet diameter (*d*_*o*_)	Chimney diameter (*d*_*ch*_) m	Chimney height (*h*_*ch*_), m	Collector diameter (*d*_*g*_*)*, m	Area ratio (*A*_*o*_/*A*_*ch*_)	Chimney outlet diametric ratio (*d*_*o*_/*d*_*ch*_)
M-1	10.16	10.16	194.6	244	1	1.0
M-21	15.24	10.16	194.6	244	2.25	1.5
M-22	20.32	10.16	194.6	244	4	2.0
M-23	25.4	10.16	194.6	244	6.25	2.5
M-24	30.48	10.16	194.6	244	9	3.0

#### Flow parameter assessment

**Figure 12:**
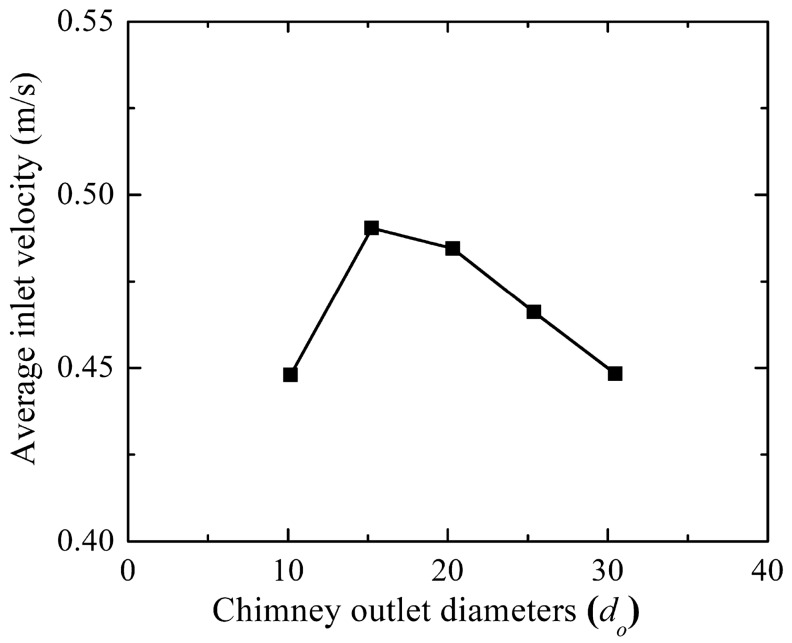
Variation of collector inlet velocity at different chimney outlet diameters.

**Figure 13:**
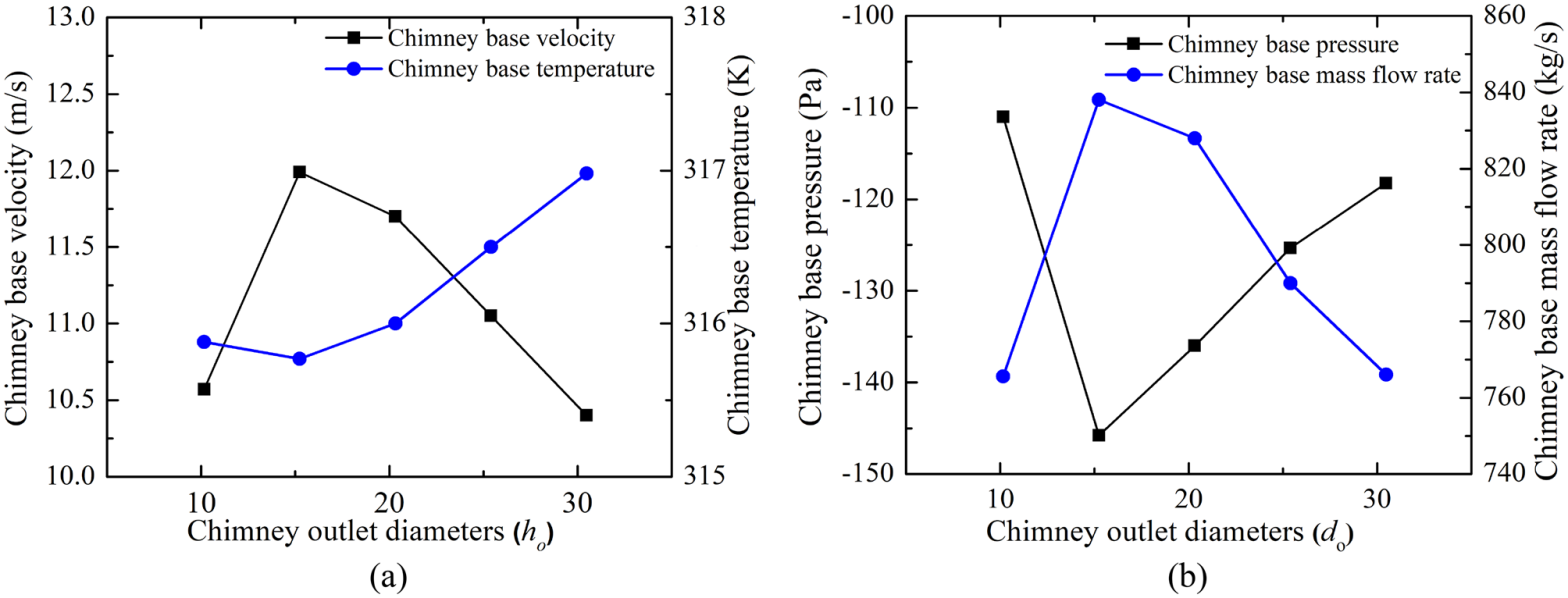
Variations of CB (**a**) velocity and temperature, (**b**) pressure, and mass flow at different chimney outlet diameters (*d*_*o*_).

**Figure 14:**
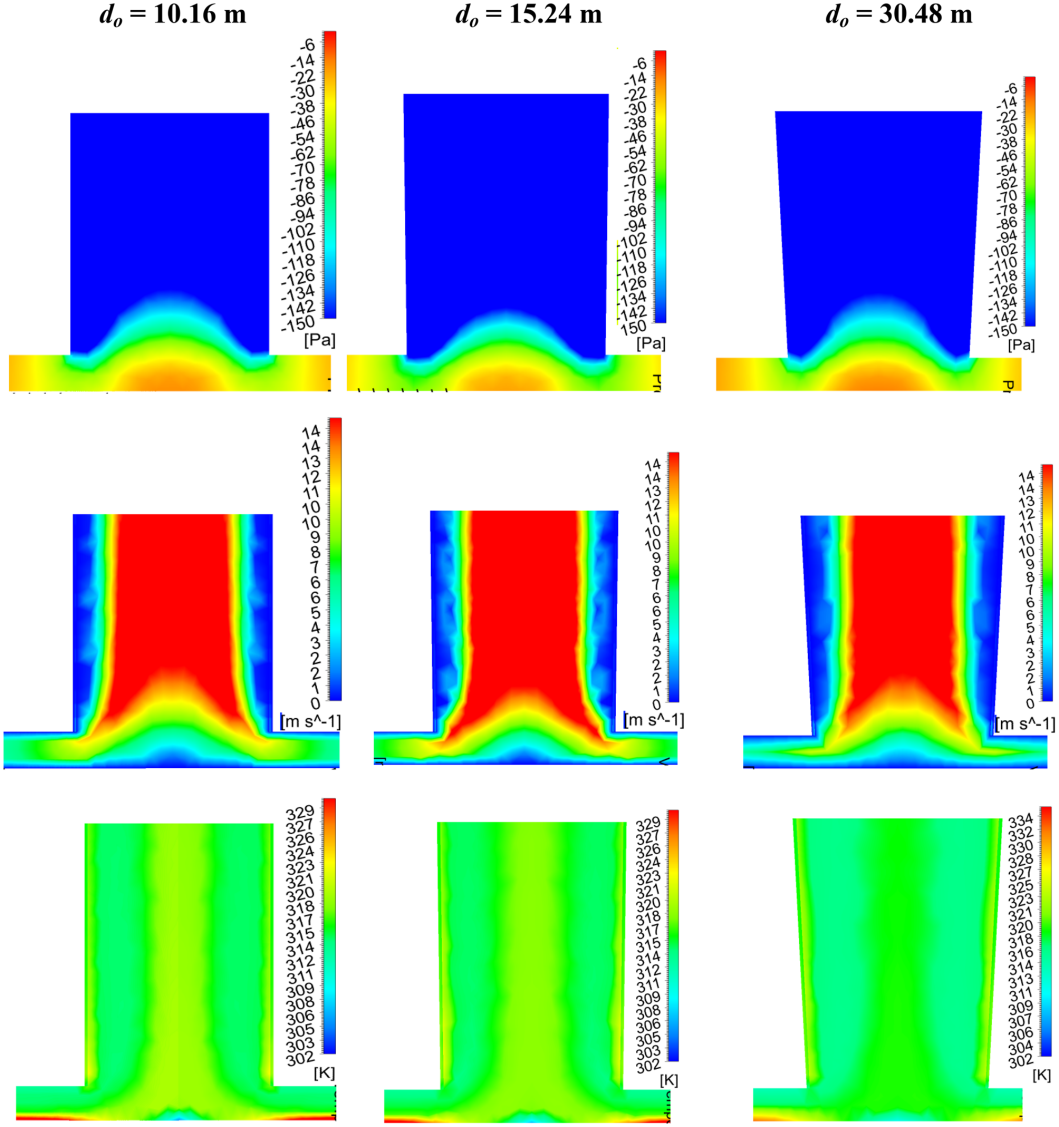
Local pressure, velocity, and temperature contour plots for the variations of chimney outlet diameters.

From the collector inlet velocity variation (as in Fig. 12), it is observed that the increase in outer diameter, *d*_*o*_ of 15.24 m (1.5 *d*_*ch*_) from the base diameter of 10.16 m enhances the flow velocity. This may lead to a rise in the suction pressure by the chimney, which in turn increases the mass flow rate. This collector inlet velocity drops further rise in the chimney outlet diameter, which may be due to the higher pressure loss in the chimney. Therefore, the area ratio of 2.25 is the optimum value to obtain the maximum inlet flow velocity. The study of CB velocity, pressure, temperature, and mass flow is illustrated for the considered chimney divergence models M-21 to 24 as shown in Fig. 13. As *h*_*i*_ and *d*_*ch*_ are fixed, the same pattern of flow velocity is observed. The magnitude of velocity is different for different areas. Due to the nozzle action, suction pressure rises, mass flow rises and the corresponding air temperature drops at *d*_*o*_ = 1.5*d*_*ch*._ Further, increase in the divergence, suction pressure drops less which lessens the mass flow rate, however, the air temperature rises as depicted. The flow contours are illustrated for *d*_*o*_ of 10.16 m, 15.24 m, and 30.48 m (1.0*d*_*ch*_, 1.5 *d*_*ch*_ and 3.0*d*_*ch*_) in Fig. 14. It reveals the optimum velocity, pressure, and temperature at chimney outlet diameter of 15.24 m.

#### Performance assessment

**Figure 15:**
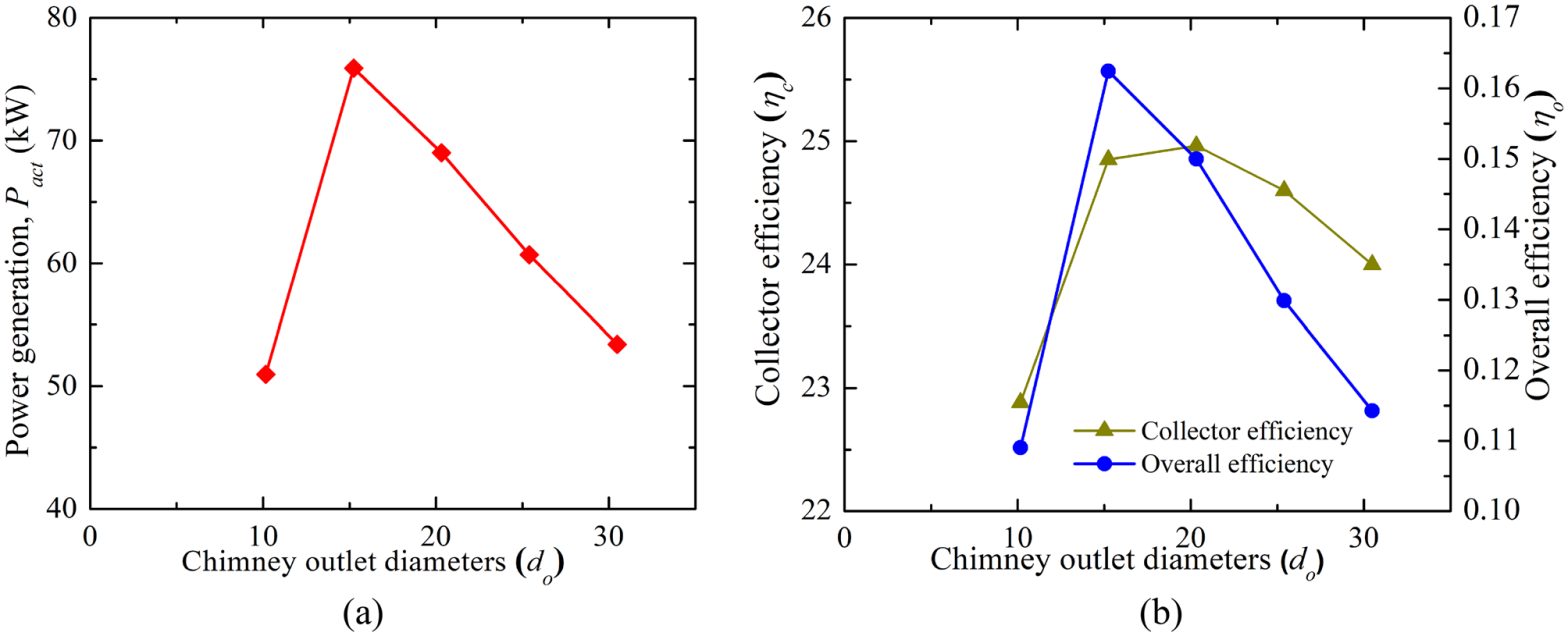
Variation of (**a**) power production and (**b**) collector efficiency (*η*_*c*_) and overall efficiency (*η*_*o*_) for the different chimney outlet diameters (*d*_*o*_).

This study reveals the power generation and efficiencies variation for the different chimney inlet diameters (*d*_*ch*_), all having an optimum value at 15.24 m (Area ratio: 2.25) outer chimney diameter (as shown in Fig. 15). Earlier studies reveal the area ratio of 10.0^20^, 4.0^25^. Here, the power generation rises to 75.91 kW, which is ~ 1.5 times more than the Manzaranes plant, corresponding efficiency is 0.16%. Comparing the effect of *h*_*i*_ and *d*_*ch*_, the impact of divergence is not effective and it requires some additional cost.

### Combined effect of collector inlet (*h*_*i*_) and chimney diameter (*d*_*ch*_)

**Figure 16:**
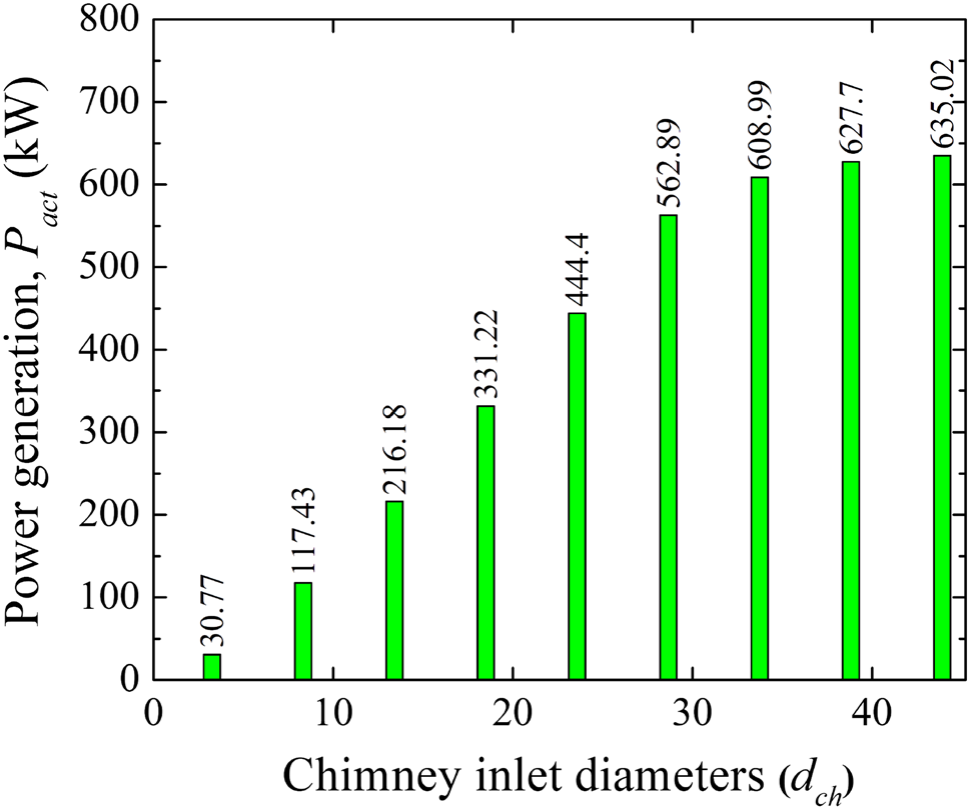
Variation of power generation (*P*_*act*_) at different model plants.

The aforesaid comparative assessment for a reduction in collector inlet height (*h*_*i*_), rise in chimney diameter (*d*_*ch*_), and chimney divergence (*d*_*o*_) shows the performance improvement of the solar chimney power plant. The lowering of chimney inlet height (*h*_*i*_) does not require any hike in the initial investment cost, whereas the chimney diameter (*d*_*ch*_) and divergence (*d*_*o*_) need a rise in the initial installation cost. The power generation rise is more for a reduction in *h*_*i*_ and rises in the chimney diameter* d*_*ch*_. Therefore few models are chosen for the study at *h*_*i*_ of 0.2 m and for different chimney diameters, 5.08 m to 45.72 m (0.5*d*_*ch*_ to 4.5*d*_*ch*_), as shown in Table 9 to study the superior performance of the SCCP. The remarkable power rise has been noted as illustrated in Fig. 16. At a lower value of *h*_*i*_ of 0.2 m, the power generation with a chimney diameter of 1.5 *d*_*ch*_ is 216.18 kW (which is 4.23 times of the Manzaranes plant), 2.0 *d*_*ch*_ is 331.22 kW (which is 6.49 times of the Manzaranes plant), 2.5 *d*_*ch*_ is 444.40 kW (which is 8.71 times of the Manzaranes plant), 3.0 *d*_*ch*_ is 562.89 kW (which is 11.03 times of the Manzaranes plant), 3.5 *d*_*ch*_ is 608.99 kW (which is 11.94 times of the Manzaranes plant), 4.0 *d*_*ch*_ is 627.7 kW (which is 12.3 times of the Manzaranes plant), 4.5 *d*_*ch*_ is 635.02 kW (which 12.45 times of the Manzaranes plant). Therefore, it summarizes that this combined model could be an alternative design for performance improvement,

**Table 9:** Models in variations of chimney diameters (*d*_*ch*_).

Model	Collector inlet (*h*_*i*_),m	Chimney diameter (*d*_*ch*_), m	Chimney height (*h*_*ch*_), m	Collector diameter (*d*_*g*_*)*, m	Chimney diametric ratio
M-25	0.2	5.08	194.6	244	0.5
M-9	0.2	10.16	194.6	244	1.0
M-26	0.2	15.24	194.6	244	1.5
M-27	0.2	20.32	194.6	244	2.0
M-28	0.2	25.4	194.6	244	2.5
M-29	0.2	30.48	194.6	244	3.0
M-30	0.2	35.56	194.6	244	3.5
M-31	0.2	40.64	194.6	244	4.0
M-32	0.2	45.72	194.6	244	4.5

### Artificial neural network (ANN) for performance analyses

**Figure 17:**
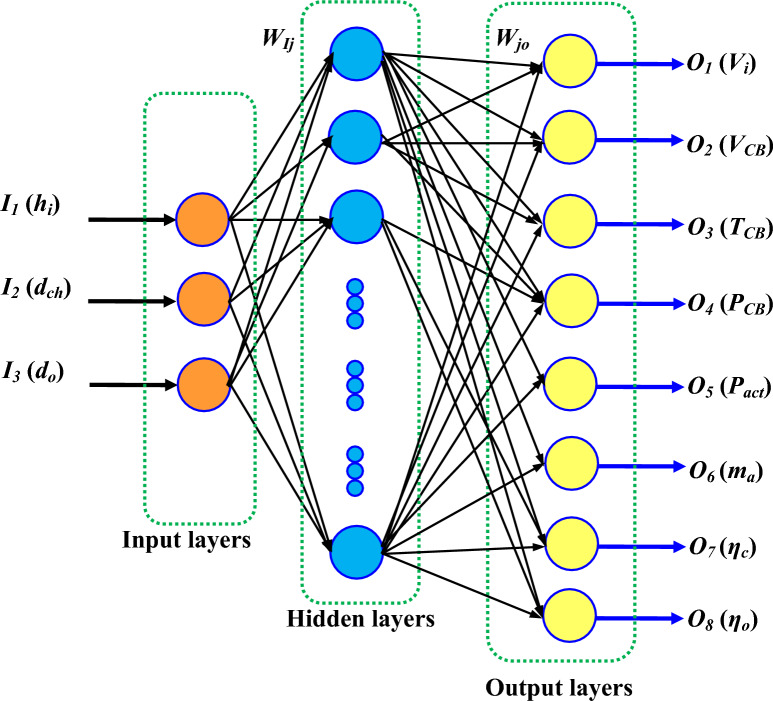
Architecture of three layers ANN with three layers namely input layer, hidden layer and output layer.

Today’s, artificial neural networks (ANN) play an important role in predicting the outputs. An application of artificial neural networks has been noted in solar heating systems^66,67^. The performance of the Solar chimney power plant is predicted by Amirkhani et al.^68^, Fadaei et al.^69^. A significant number of inputs and outputs are required in order to create an ANN model. In the present study, ANN is used to predict the flow and performance parameters by using input data such as collector inlet height (*h*_*i*_), chimney diameter (*d*_*ch*_), and chimney divergence (*d*_*o*_). Chimney height, collector diameter, and solar intensity remained constant. The output variables are inlet velocity (*V*_*i*_), chimney base velocity (*V*_*CB*_), chimney base temperature (*T*_*CB*_), chimney base pressure (*p*_*CB*_), mass flow rate (*m*_*a*_), Power generation (*P*_*act*_), collector efficiency (*η*_*c*_) and overall efficiency (*η*_*o*_). The general architecture of the three layers of ANN is shown in Fig. 17. The 1st layer is the input layer, the 2nd layer is the hidden layer, and 3rd layer i.e. the last layer is the output layer. Each neuron in the hidden layer is assigned weight (*W*_*Ij*_) and bias (*B*_*j*_). Each neuron in the output layer is assigned weight (*W*_*jO*_) and bias (*B*_*O*_). In our design, the input layer consists of three neurons namely, I_1_(*h*_*i*_), I_2_(*d*_*ch*_), and I_3_(*d*_*o*_), and the output layer consists of eight neurons namely, O_1_(*V*_*i*_), O_2_(*V*_*CB*_), O_3_(*T*_*CB*_), O_4_ (*p*_*CB*_), O_5_(*P*_*act*_), O_6_ (*m*_*a*_), O_7_(*η*_*c*_), and O_8_(*η*_*o*_).

#### Forward and backward propagation

The forward propagation method works on the input layer to the hidden layer and the hidden layer to the output layer. This method is used to calculate the output of each neuron in the hidden layer and output layer^70, 71^. It starts operation by initializing a random weight and bias. Then calculates the output of each neuron in the hidden and output layers using the activation function. There are different types of activation functions such as Thresholds, Sigmoid, Tanh, ReLu, and tansig. Here we used the tansig activation function to calculate the output in the hidden and output layers. The activation function (tansig) is given below:

10 $$F(z) = \frac{2}{{1 + e^{ - 2z} }} - 1,$$

where $z = \sum\limits_{i = 1}^{n} {I_{i} } W_{i} + B$, with I is the input values, *W* is the transpose of the weight between input and hidden layers and *B* is the bias vector in a different layer. Using the above equation we can predict output from the output layer. Now at each output, the error will be calculated using the following formula:

11 $${\text{Error}}_{{{\text{total}}}} = \sum\limits_{1}^{8} {\frac{1}{8}(Target_{output} - {\text{Predicted}}_{output} )^{2} } .$$

If the error is large then we use the backward propagation method to correct the weights. The backward propagation method works in the output layer to the hidden layer and the hidden layer to the input layer. In different layers weights are updated using the following equation:

12 $$W^{*} = W - \lambda \frac{{\partial {\text{Error}}_{{{\text{total}}}} }}{\partial W},$$

where *λ* is the learning rate of the design, which signifies how quickly the design predicts the target output. *W* is the weight vector in different layers. So using the above equation weights will be updated by the backward propagation method.

#### Determination of the number of neurons in the hidden layer

**Figure 18:**
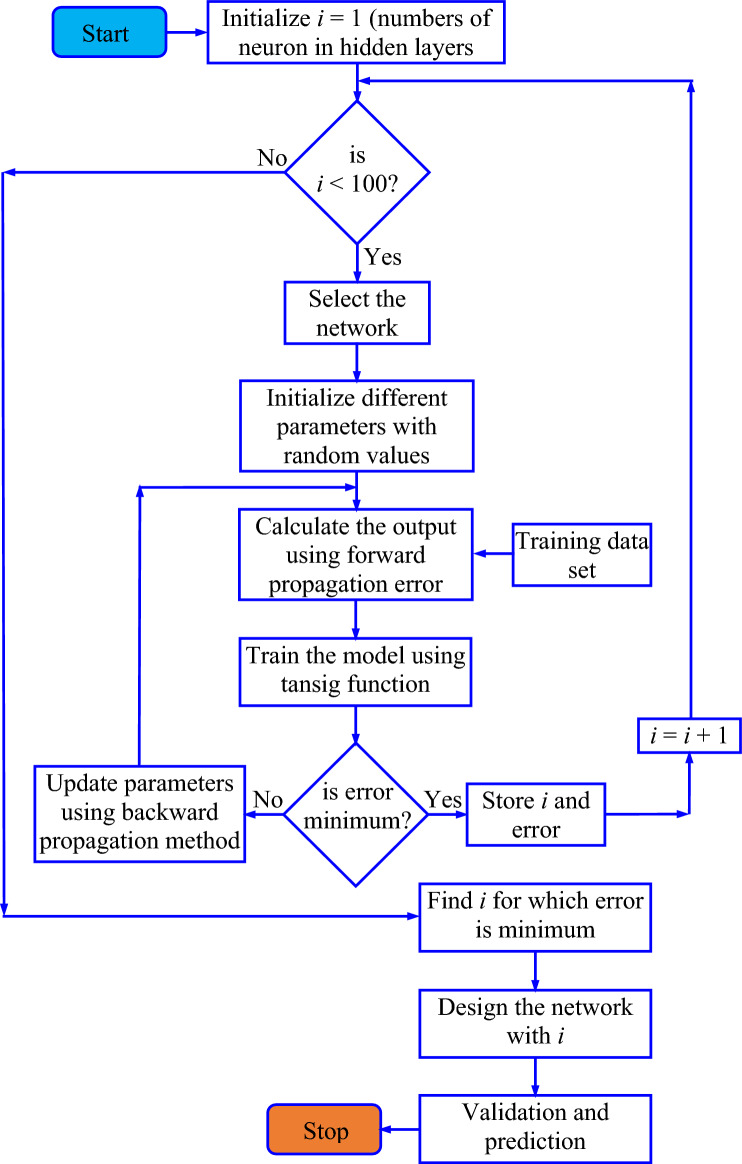
Flow chart to determine the number of neurons in the hidden layer.

To design an efficient neural network, it is necessary to know the number of neurons in the hidden layer. The following flowchart (as shown in Fig. 18) is utilized to determine the number of neurons in the hidden layer.

**Figure 19:**
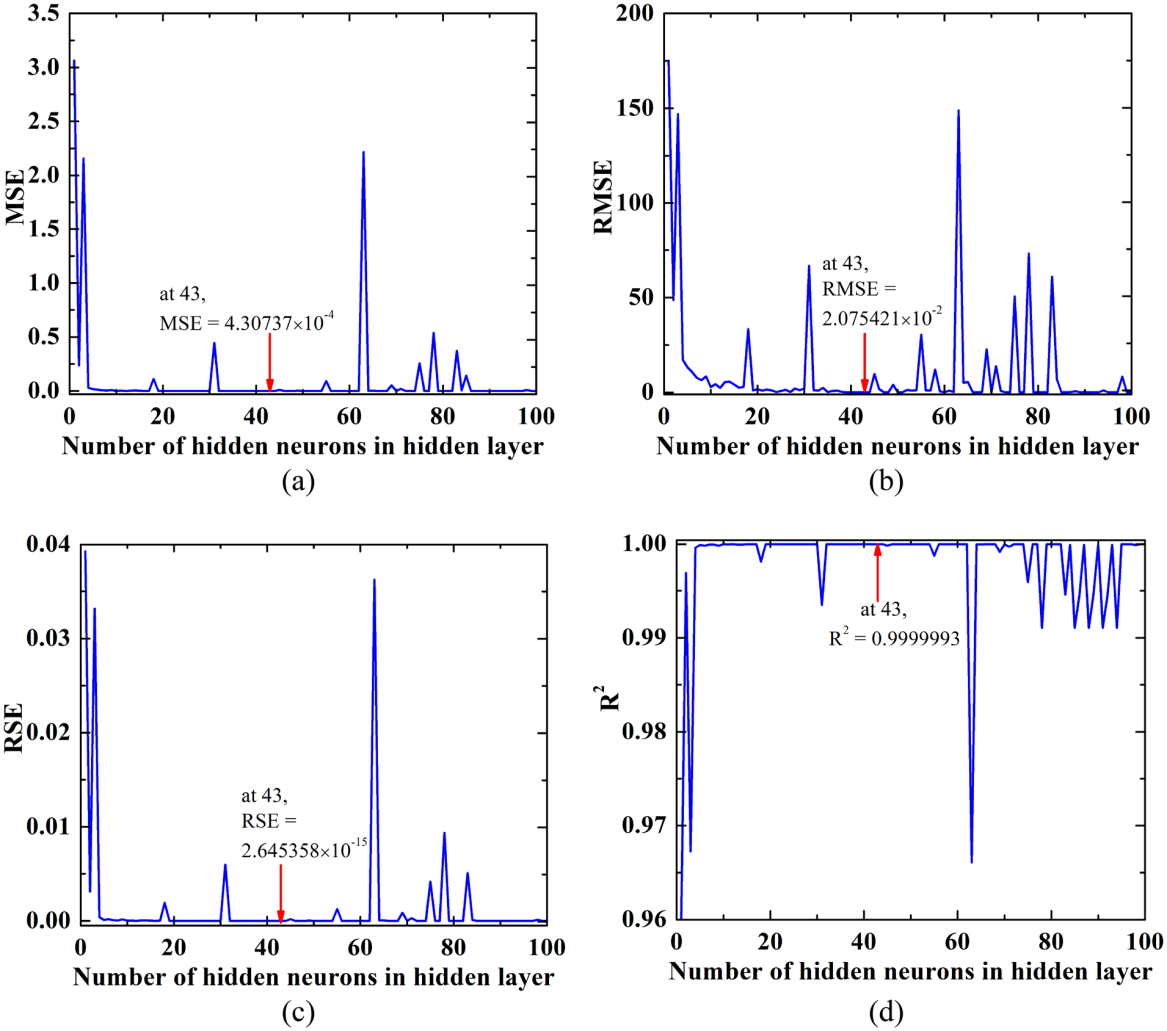
Error with number of neurons in the hidden (**a**) Mean Square Error (MSE), (**b**) Root Mean Square Error (RMSE), (**c**) Relative Square Error (RSE), and (**d**) Correlation Coefficient (R^2^).

To determine the number of neurons in the hidden layer, we have calculated mean square error (MSE), root mean square error (RMSE), relative square error (RSE), and correlation coefficient (R^2^). The following formulas are used for the calculation of various error parameters.

13 $${\text{Mean square error }}\left( {{\text{MSE}}} \right) = \frac{1}{n}\sum\limits_{k = 1}^{n} {(y_{train} - y_{{{\text{true}}}} )^{2} }$$

14 $${\text{Root mean square error }}\left( {{\text{RMSE}}} \right) = \sqrt {\frac{1}{n}\sum\limits_{k = 1}^{n} {(y_{train} - y_{{{\text{true}}}} )^{2} } }$$

15 $${\text{Relative square error }}\left( {{\text{RSE}}} \right) = \frac{{\sum\limits_{k = 1}^{n} {(y_{train} - y_{{{\text{true}}}} )^{2} } }}{{\sum\limits_{k = 1}^{n} {(\overline{{y_{train} }} - y_{{{\text{true}}}} )^{2} } }}$$

16 $${\text{Correlation coefficient }}\left( {{\text{R}}^{{2}} } \right) = 1 - \frac{{\sum\limits_{k = 1}^{n} {(y_{train} - y_{{{\text{true}}}} )^{2} } }}{{\sum\limits_{k = 1}^{n} {(y_{train} - \overline{{y_{{{\text{true}}}} }} )^{2} } }}$$

where *n* is the total number of data sets, $y_{{{\text{true}}}}$ is the actual (target) output, $\overline{{y_{{{\text{true}}}} }}$ is the mean of the actual (target) output, and $y_{train}$ is the ANN output (predicted) after training the model. We have plotted the MSE, RMSE, RSE, and R^2^ with different values of the number of neurons in the hidden layer as shown in Fig. 19. Our goal is to find the number of neurons for which MSE, RMSE, and RSE are minimum and correlation coefficient close to 1. To ascertain the optimal number of neurons for our hidden layer, we relied on a flowchart (Fig. 18) and computed various error metrics, including MSE, RMSE, RSE, and R^2^ (Eqs. 13–16). We aimed to identify the neuron count that simultaneously minimized MSE, RMSE, and RSE while maximizing R^2^. As depicted in Fig. 19, the hidden layer comprising 43 neurons exhibited the most favorable outcomes, yielding the lowest values for MSE, RMSE, and RSE (approximately 0.000430737, 0.02075421, and 2.645358 × 10^–15^, respectively). Additionally, the correlation coefficient approached 1 (0.9999993). As a result, we proceeded to create a network with a hidden layer comprising 43 neurons.

#### Network design and predicted results

**Figure 20:**
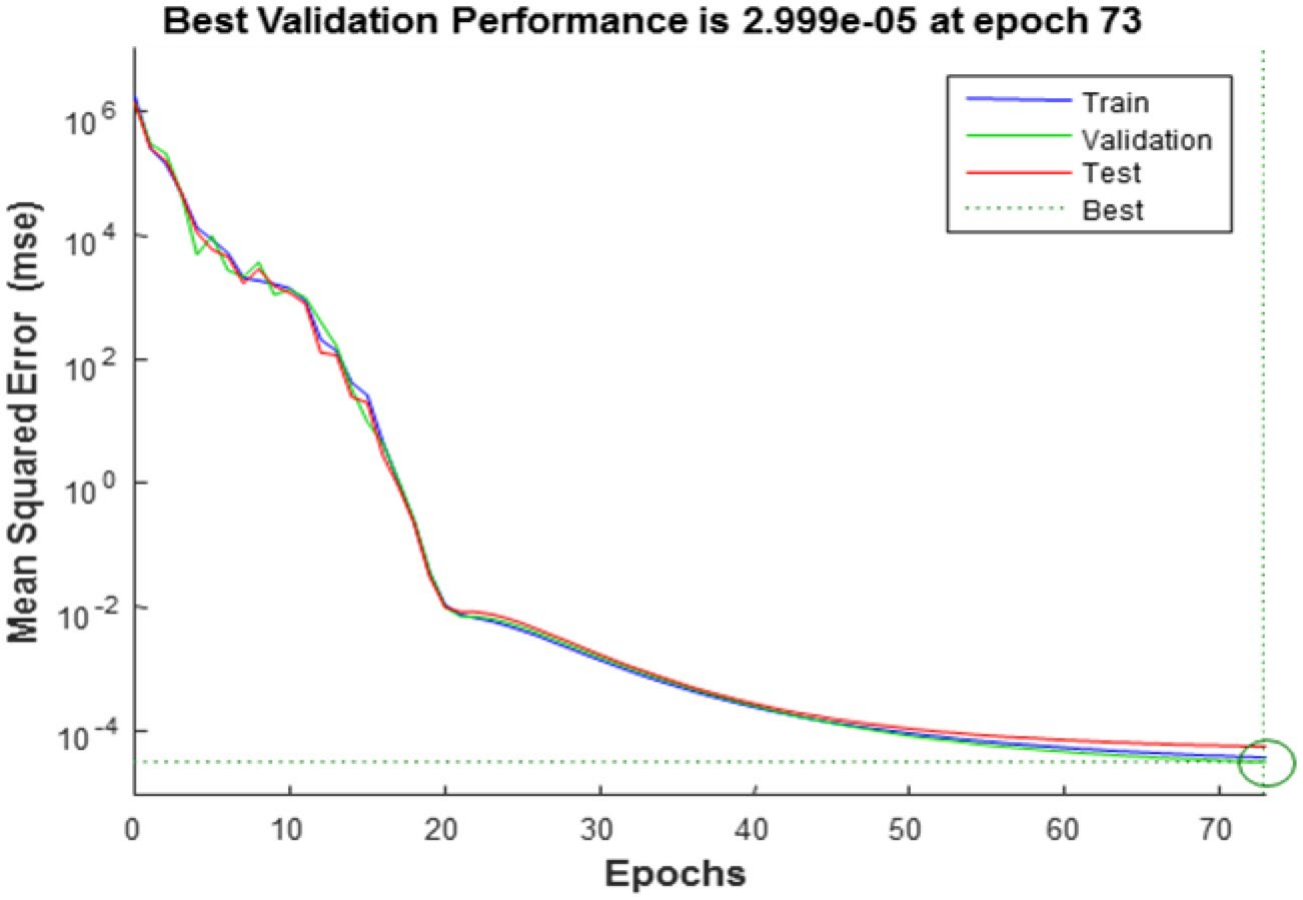
Error with number of iterations (Epochs).

Now we have designed the artificial neural network with 43 neurons in the hidden layer. To mitigate overfitting, we partitioned our dataset into three segments: the test set, the training set, and the validation set. The validation set played a pivotal role in monitoring our model's performance during training. If the model performs well on the training set but poorly on the validation set, it may be overfitting. We have trained the neural network using the training dataset. To reduce error, the network’s weight function is corrected by the back propagation method. The validation dataset is used to calculate network generalizations, and we stopped training when generalizations did not improve. With the use of the testing dataset, the network’s performance is assessed both during and after training. In this approach, the training process uses 85% of the whole data set, testing uses 10% of the data set, and assessing network performance uses the remaining dataset. The change of MSE for training, validation, and testing data sets with the number of epochs is depicted in Fig. 20. Figure 20 shows that the best validation performance, 2.999 × 10^–5^ at epoch 73, was achieved. We stopped the training process after epoch 73 because the number of iterations increased and the errors increased, which may indicate overfitting.

**Figure 21:**
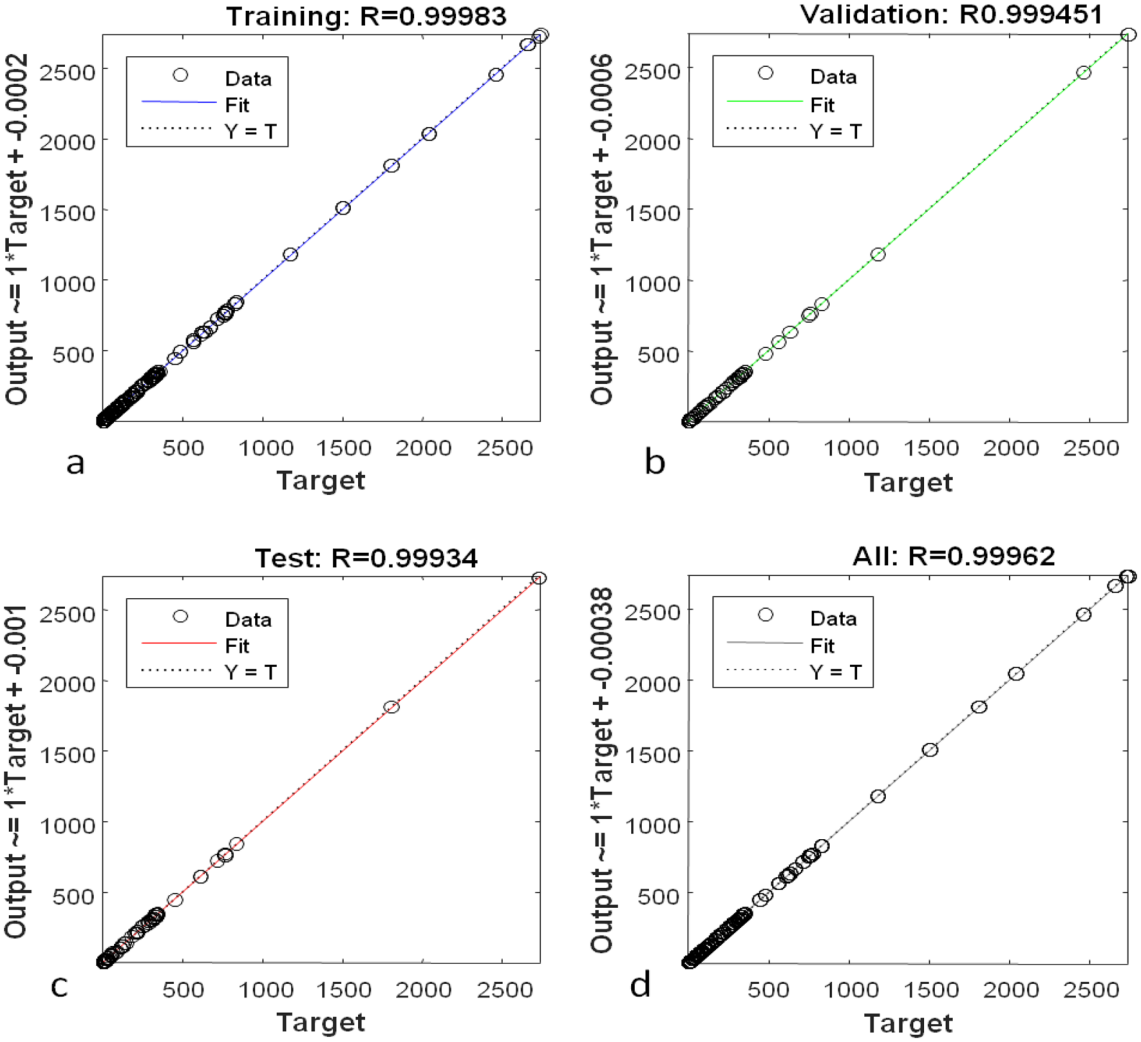
Variation of error for various data sets of (**a**) training data set, (**b**) validation data set, (**c**) test data set, and (**d**) overall data set.

Figure 21 displays the error for various data sets (training, validation, testing, and overall). The results show that the error factor is closer to 1, demonstrating the neural network's ability to fully associate the input data set with the model data set. The values error in the training data set, validation data set, test dataset, and overall data set are 0.99983, 0.999451, 0.99934, and 0.99962 respectively. This shows that the results are reasonably good for the designed neural network.

**Figure 22:**
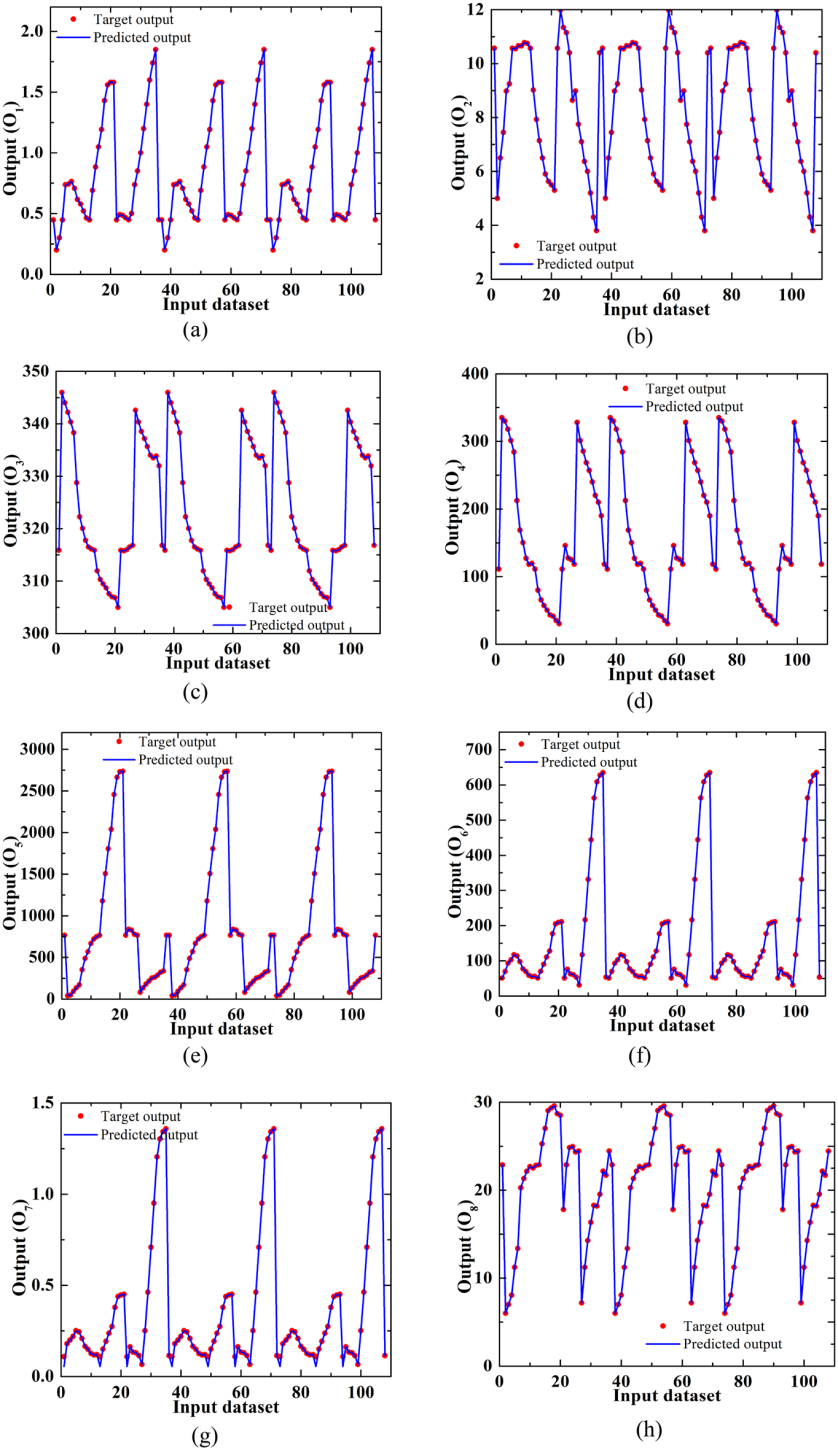
Distribution of output of the target and predicted values for different data sets at the output (**a**) O_1_, (**b**) O_2_, (**c**) O_3_, (**d**) O_4_, (**e**) O_5_, (**f**) O_6_, (**g**) O_7,_ and (**h**) O_8_.

With this trained network, we have now calculated expected output values for several data sets. Figure 22 displays the distribution of goals and anticipated values at the outputs O_1_, to O_8_ for several data sets.

It can be seen from the aforementioned Fig. 22 that the 43 neurons constructed neural network accurately predicts the output values. Therefore, we may anticipate output values for an unknown data set using this trained neural network.

To predict the developed power (*P*_*act*_) of SCPP as a function of collector inlet height (*h*_*i*_), chimney inlet diameter (*d*_*ch*_), and chimney outlet diameter (*d*_*o*_) for the constant collector diameter and same irradiation, regression analysis has been carried out using MATLAB code with the inbuilt function of *nlinfit*. The correlation is expressed as

21 $$P_{act} = a_{1} + a_{2} h_{i} + a_{3} d_{ch} + a_{4} d_{o} + a_{5} h_{i} d_{ch} + a_{6} h_{i} d_{o} + a_{7} d_{ch} d_{o} + a_{8} h_{i}^{0.1} + a_{9} d_{ch}^{2} + a_{10} d_{o}^{2}$$

where the variables *a*_*n*_ are given as *a*_*1*_ − 201.8916679297755, *a*_*2*_ − 131.9161514753086, *a*_*3*_ 14.0151979342754, *a*_*4*_ − 11.4489669355259, *a*_*5*_ − 5.2534078301303, *a*_*6*_ 8.8785124787426, *a*_*7*_ 0.9090599083684, *a*_*8*_ 316.7444829699626, *a*_*9*_ − 0.1564657170176, *a*_*10*_ − 0.3274118939252.

## Conclusion

This work attempts for enhancing the power generation of the classical Manzanares solar chimney (SC) plant by modifying the collector inlet height, chimney diameter, and its divergence to obtain the best design of the plant. The performance assessment is evaluated for the different combinations of specified geometric parameters like collector inlet (*h*_*i*_), chimney diameter (*d*_*ch*_), and chimney divergence by exit diameter (*d*_*o*_). The outcomes of the analysis are presented through the local distributions (pressure, velocity, temperature, mass flow) and performance parameters (power generation and efficiencies). Furthermore, an attempt has been made to utilize an artificial neural network (ANN) for developing the performance model, which could be very helpful for the designer of the prototype design.

This study reveals that the power developed by the turbine rises always at the lowering collector inlet height of the classical Manzaranes plant. The maximum power lies at *h*_*i*_ = 0.2 m, which is *P*_*act*_ = 117.42 kW. The maximum efficiency occurs at the same location, which is 0.25%. Therefore, the power generation rises from 51 kW to 117.42 kW (~ 2.3 times) without increasing any additional installation cost. As *h*_*i*_ reduces, the chimney base velocity drops, suction pressure increases, the temperature rises, and mass flow drops. The optimum collector inlet velocity occurs at *h*_*i*_ = 0.2 m.

A rise in the chimney diameter (*d*_*ch*_) lowers the chimney base velocity, and suction pressure, both. In spite of these, the power generation by the turbine rises, and the flow becomes stagnant after the chimney diameter of 45.72 m (corresponding slenderness ratio: 4.5). No significant alteration in the collector inlet velocity is noted after this diameter. The maximum power generation at this chimney diameter is 209 kW, which is ~ 4 times than the Manzaranes plant, corresponding efficiency is 0.44%. However, this rise in the chimney diameter requires a higher initial investment cost.

The study on chimney divergence reveals that the power production, collector as well as overall efficiency, and collector inlet velocity, all have an optimum value at 15.24 m (area ratio: 2.25) outer chimney diameter. Then, the power generation rises to 75.91 kW which is ~ 1.5 times more than the Manzaranes plant, the corresponding efficiency is 0.16%.

At a lower *h*_*i*_ of 0.2 m, the power generation with the chimney diameter of 1.5 *d*_*ch*_ is 216.18 kW, 2.0 *d*_*ch*_ is 331.22 kW, 2.5 *d*_*ch*_ is 444.40 kW, 3.0 *d*_*ch*_ is 562 kW, 3.5 *d*_*ch*_ is 608.99 kW, 4.0 *d*_*ch*_ is 627.7 kW, 4.5 *d*_*ch*_ is 635.02 kW (12.45 times of Manzaranes plant).

An artificial neural network (ANN) is utilized for developing performance modeling to predict the output parameters.

## Data Availability

The datasets used and/or analysed during the current study available from the corresponding author on reasonable request.

## List of symbols

*A*_*ch*_: Chimney area, *m*^*2*^
*A*_*coll*_: Area of collector, *m*^*2*^
*c*_*p*_: Specific heat of air, *kJ/kgK*
*d*_*ch*_: Chimney diameter, *m*
*h*_*i*_: Inlet height of collector
*d*_*c*_: Diameter of collector, *m*
*d*_*g*_: Diameter of ground, *m*
*d*_*o*_: Chimney exit diameter, *m*
*h*_*ch*_: Chimney length, *m*
Gr: Grashof number
*I*: Solar irradiation, *W/m*^*2*^
*L*: Characteristic length, *m*
*m*_*a*_: Mass flow rate, *kg/s*
*p*: Pressure, *Pa*
Pr: Prandtl number
*Q*: Volume flow rate, *m*^*3*^*/s*
Ra: Rayleigh number
*T*: Temperature, *K*
*V*: Velocity, *m/s*
*P*_*act*_: Electrical power developed by generator, W
*u*_*i*_: Velocity vector, m/s

## Greek symbols

$$\alpha$$: Thermal diffusivity, m^2^/s
$\beta$: Thermal expansion coefficient, 1/K
$\mu$: Dynamic viscosity, kg/ms
$$\nu$$: Kinematic viscosity, m^2^/s
$$\eta_{c}$$: Collector efficiency, %
$$\eta_{o}$$: Overall efficiency, %
$\rho_{air}$: Ambient air density, kg/m^3^

## Subscripts

*c*: Collector
*ch*: Chimney
*CB*: Chimney base
*eff*: Efficiency
*in*: Inlet
*o*: Overall
*out*: Outlet
